# Pain Sensitivity, Psychological Factors, and Muscle Function in Male Athletes With Long‐Standing Groin Pain and Matched Controls

**DOI:** 10.1111/sms.70334

**Published:** 2026-07-06

**Authors:** Mathias Fabricius Nielsen, Lasse Ishøi, Mikkel Bek Clausen, Carsten Juhl, Shellie Boudreau, Thomas Graven‐Nielsen, Per Hölmich, Kristian Thorborg

**Affiliations:** ^1^ Sports Orthopedic Research Center – Copenhagen (SORC‐C), Department of Orthopedic Surgery Copenhagen University Hospital – Amager‐Hvidovre Hvidovre Denmark; ^2^ Human Performance Department, Right to Dream Farum Denmark; ^3^ Department of Midwifery, Physiotherapy, Occupational Therapy and Psychomotor Therapy, Faculty of Health University College Copenhagen Copenhagen Denmark; ^4^ Department of Sports Science and Clinical Biomechanics (IOB) University of Southern Denmark Odense Denmark; ^5^ Department of Physiotherapy and Occupational Therapy Copenhagen University Hospital Herlev and Gentofte Denmark; ^6^ Center for Neuroplasticity and Pain (CNAP), Department of Health Science and Technology Aalborg University Aalborg Denmark; ^7^ Aglance Solutions ApS Aalborg Denmark

**Keywords:** adductor‐related, football, pressure pain, quantitative sensory testing, rate of force

## Abstract

Long‐standing groin pain in athletes is often managed as a localized musculoskeletal tissue injury. Treatment‐resistant symptoms and disability after return to sport suggest that other factors may also contribute. This study compared pain sensitivity, psychological factors, and muscle function between male athletes with long‐standing groin pain (*N* = 20) and sex‐, age‐, and sports‐matched controls (*N* = 20). Pressure‐pain thresholds were assessed with a handheld algometer at the adductor longus muscle and origin, iliopsoas tendon, pubic bone, pubic symphysis, gluteus maximus muscle, and elbow. Pain detection and tolerance thresholds, temporal summation of pain, and conditioned pain modulation were assessed at the lower leg with cuff‐pressure algometry. Catastrophizing, fear of movement, pain self‐efficacy, depression, anxiety, stress, loneliness, and sleep were assessed. Maximal and explosive hip‐adduction and ‐abduction strength were assessed. Between‐group effects were expressed as Cohen's *d* or rank‐biserial correlation *r*. Athletes with long‐standing groin pain showed lower pressure‐pain thresholds on the most painful side (*d* = 0.7–1.2), and lower pain detection threshold (*d* = 0.7). They reported more pain catastrophizing (*r* = 0.6), fear of movement (*r* = 0.4–0.5), depressive symptoms (*r* = 0.3–0.4), and lower pain self‐efficacy (*r* = 0.6). Explosive strength was lower during both hip‐adduction (*d* = 0.8) and hip‐abduction (*d* = 0.7–0.9). At the group level, male athletes with long‐standing groin pain showed altered pain sensitivity, adverse psychological factors, and lower explosive hip strength, despite inter‐individual heterogeneity in these factors. Athletes with these characteristics may require a more biopsychosocial management than the traditional approach.

## 1 Introduction

Long‐standing groin pain is a common condition in male football players [1]. It lasts from 6 weeks to more than 12 months [2] and cause significant reductions in physical function, football participation, and quality of life [1, 3]. Symptoms most commonly present as unilateral pain at the adductor longus origin [4], but pain radiating throughout the groin, medial thigh, anterior hip, and lower abdomen is not uncommon [4]. Based on symptoms and clinical findings, long‐standing groin pain is classified into the clinical entities adductor, iliopsoas, inguinal, pubic, and/or hip‐related groin pain [5], with adductor‐related groin pain being the most prevalent entity [4, 5]. However, multiple coexisting entities are common [4, 6], and are associated with higher pain intensity [6], greater disability [6]. In addition, up to one‐third of athletes report bilateral symptoms [4, 6], which are linked to a wider pain distribution [4], poorer treatment outcomes [7] and a higher likelihood of multiple entities [4, 6], higher pain intensity [6], and worse disability [6].

Current management targets the distinct structures of each clinical entity and physical deficits with progressive exercises and adjunct therapies [8]. Following such strategies, between 14% and 50% of athletes do not return to pre‐injury sport and performance [7, 9], and many athletes continue to report disability and physical deficits even after returning to sport [3, 10]. These observations indicate that factors contributing to disability and return to sport remain unexplored and unaddressed [3, 8]. Guided by a biopsychosocial framework, the International Olympic Committee (IOC) recently advocated for assessing all potentially relevant neurophysiological, psychological, and biomechanical factors when managing athletes with long‐standing pain [11, 12].

Pain sensitivity [11] is a novel and important neurophysiological factor of pain, as altered pain sensitivity (e.g., hyperalgesia) may contribute to amplified pain intensity and pain areas independent of local structural pathology [13, 14]. Despite its relevance, pain sensitivity is rarely assessed with standardized methods in athletes with long‐standing groin pain; one study showed local mechanical hyperalgesia at the adductor longus tendon in male athletes with mixed clinical entities of groin pain [15]. Another study of patients with hip‐related groin pain reported facilitated temporal summation of pain at sites remote from the hip, and found that facilitated temporal summation was associated with higher pain intensity before and after hip arthroscopy [16].

Psychological factors are well‐established as important for both pain experience [11], and rehabilitation [17], however such factors remain underexplored in male athletes with long‐standing groin pain. Commonly used outcome measures, such as the International Hip Outcome Tool‐33 [18] and Hip and Groin Outcome Score (HAGOS) [19], include items on fear avoidance behavior, sleep disturbances, negative mood, and frustration [18, 19], but these psychological components are lost within summary scores. A study showed elevated levels of pain catastrophizing in athletes with long‐standing pubic‐ and/or adductor‐related groin pain [10], while several studies showed that poorer mental health is associated with worse pain, function, and treatment outcomes in patients with hip‐related groin pain [20].

Long‐standing pain can also influence neurophysiological and biomechanical factors that regulates muscle function [12]. Athletes with long‐standing groin pain have lower maximal eccentric and isometric hip adduction strength compared to healthy athletes [21]. However, improvement in these strength measures explain only a small proportion of disability improvements after treatment [3]. Explosive strength (e.g., rate of force/torque development) is an aspect of muscle function that seem more sensitive to pain and neurophysiological changes than isometric strength [22]. Explosive strength is critical for sprinting and rapid sport‐specific movements [22, 23], however, it has not been investigated in athletes with long‐standing groin pain.

Our study aimed to characterize pain sensitivity, psychological factors, and muscle function (maximal and explosive strength) in male athletes with long‐standing groin pain compared to sex‐, age‐, and sports‐matched asymptomatic controls.

## 2 Methods

This study was designed as an explorative cross‐sectional case–control study with a flat outcome structure. Consequently, no single primary outcome was predefined across the three outcome domains: pain sensitivity, psychological factors, and muscle function. The study was approved by the Danish Capital Region ethics committee (H‐20055130) and conducted in accordance with the Declaration of Helsinki. Reporting follows STROBE guidelines. Data were collected from November 2021 to April 2024 at the Sports Orthopedic Research Center—Copenhagen (Copenhagen University Hospital Amager‐Hvidovre, Denmark). All athletes gave written informed consent. As reimbursement, athletes with long‐standing groin pain received a post‐study treatment consultation, while asymptomatic controls received 300 Danish Krones.

### 2.1 Participants

Male athletes (18–35 years, Danish speaking) from the Capital Region/Zealand Denmark were recruited via social media, online ads, and football clubs. Symptomatic and asymptomatic athletes were group‐matched by sex, age (±1–2 years), and sport (type).

Inclusion criteria for symptomatic athletes were ≥ 3 h/week sports activity and groin pain lasting ≥ 3 months, and a clinical classification of adductor‐related groin pain. Symptomatic athletes were not excluded based on the presence of coexisting iliopsoas‐, inguinal‐, and/or pubic‐related groin pain, as these entities commonly coexist with adductor‐related groin pain [4, 6]. However, athletes were excluded if they were not classified with adductor‐related groin pain.

Additional exclusion criteria were hip‐joint related groin pain (a positive Flexion‐Adduction‐Internal Rotation (FADIR) test and a positive diagnostic hip injection), hip dysplasia (lateral center edge angle < 20°), borderline hip dysplasia (lateral center edge angle = 20°–25°), pre‐existing hip osteoarthritis (lateral joint space width < 3 mm), Perthes' disease, slipped upper femoral epiphysis, avascular necrosis, acetabular fracture, hip dislocation, femoral neck fracture, stress fracture, referred pain from facet or sacroiliac joints, previous hip surgery, serious pathology, competing diseases, or a reduced sports‐activity for reasons other than groin pain.

Inclusion criteria for asymptomatic athletes were ≥ 3 h/week sports activity, no hip and groin pain, no musculoskeletal pain or injuries in the past 3 months, and no groin pain during the Copenhagen 5‐s squeeze test [24].

### 2.2 Study Procedures

After telephone pre‐screening, eligible athletes attended in‐person screening, standardized clinical and radiographic examinations, and one experimental session (Table S1). A male physiotherapist (MFN) with 5 years of experience conducted the pre‐screening, in‐person screening, clinical examination of asymptomatic athletes, and the experimental testing.

A male orthopedic surgeon specializing in groin pain (PH), with 33 years of experience, assessed study eligibility of symptomatic athletes by applying predefined inclusion and exclusion criteria during the clinical examination, including classification of groin pain and review of radiographs.

Following full inclusion and completion of experimental testing, an independent musculoskeletal radiologist re‐evaluated all radiographs to generate a complete set of descriptive data on hip‐ and pubic‐related radiographic parameters [25].

Due to practical constraints, the experimental assessor (MFN) was not blinded to group allocation during the experimental session. Standardized, scripted instructions were used to ensure consistent information delivery and minimize fear related to the procedures. Athletes avoided analgesics for ≥ 8 h prior to testing.

### 2.3 Clinical and Radiological Examination

The clinical examination included history taking, palpation, manual resistance testing, passive stretching, the FADIR test, and the Flexion‐Abduction‐External Rotation (FABER) test. Palpation and tests were considered positive if they reproduced the athlete's known and recognizable groin pain [6]. If the FADIR test was positive, a diagnostic hip injection was administered to classify hip‐related groin pain. Symptomatic athletes were classified with clinical entities of groin pain according to the Doha agreement [5].

Symptomatic athletes underwent anteroposterior pelvic and cross‐table lateral hip radiographic examinations. Radiographs were reviewed by the orthopedic surgeon as part of the clinical examination to screen hip‐related radiographic parameters and identify radiographic exclusion criteria [25]. Radiographs were subsequently re‐evaluated by an independent musculoskeletal radiologist for descriptive purposes (see Section 2.2) [25].

Radiographic measurements performed by the orthopedic surgeon were used for study inclusion and classification of groin pain, whereas measurements performed by the radiologist were only used for descriptive reporting in Tables 1 and A2.

**TABLE 1:** Participant characteristics.

Characteristics	Symptomatic athletes	Asymptomatic athletes
*N*	20	20
Age, years, mean (SD)	27 (6)	26 (4)
Height, cm, mean (SD)	181 (8)	184 (5)
Weight, kg, mean (SD)	77 (10)	81 (8)
BMI, mean (SD)	23 (2)	24 (3)
Sport, hours per week, mean (SD)	6.5 (3.1)	6.9 (3.3)
Sport type		
Football, *n* (%)	18 (90)	18 (90)
Volleyball, *n* (%)	1 (5)	1 (5)
Climbing, *n* (%)	1 (5)	1 (5)
Sport level^a^		
Elite, *n* (%)	3 (16)	2 (10)
Competition, *n* (%)	10 (53)	8 (40)
Leisure activity, *n* (%)	6 (32)	10 (50)
Education level^b^		
Elementary or less, *n* (%)	3 (15)	0 (0)
High School, *n* (%)	5 (25)	11 (61)
Vocational education, *n* (%)	4 (20)	0 (0)
Short higher education, *n* (%)	1 (5)	2 (11)
Medium higher education, *n* (%)	4 (20)	3 (17)
Long higher education or more, *n* (%)	3 (15)	2 (11)
Dominant leg		
Left, *n* (%)	2 (10)	4 (20)
Right, *n* (%)	18 (90)	16 (80)
Painful side		
Left, *n* (%)	4 (20)	—
Right, *n* (%)	9 (45)	—
Bilateral, *n* (%)	7 (35)	—
Most painful side		
Left, *n* (%)	1 (14)	—
Right, *n* (%)	6 (86)	—
Injury mechanism		
Acute after specific situation, *n* (%)	7 (35)	—
Gradual after specific situation, *n* (%)	6 (30)	—
Gradual and unspecific, *n* (%)	7 (35)	—
Previously treated for current groin pain, *n* (%)	13 (65)	—
Physiotherapy, unspecified, *n* (%)	7 (35)	—
Chiropractor, *n* (%)	1 (5)	—
Massage, *n* (%)	3 (20)	—
Exercises, *n* (%)	3 (20)	—
Body SDS, *n* (%)	1 (5)	—
Pain medication use, *n* (%)	3 (15)	—
Average days per week, median (IQR)	3 (2–4)	—
Types of pain medication		
Paracetamol, *n* (%)	3 (15)	—
Ibuprofen, *n* (%)	2 (10)	—
Pain duration, weeks, median (IQR)	51 (26–175)	—
Pain intensity in preceding week		
Worst pain, median (IQR)	6.5 (4.8–7.0)	—
Least pain, median (IQR)	0.5 (0.0–1.3)	—
Average pain, median (IQR)	3.0 (2.0–4.0)	—
Current pain intensity, median (IQR)	2.5 (0.0–3.3)	—
Pain intensity composite score, median (IQR)	2.0 (1.0–2.5)	—
HAGOS		
Pain, median (IQR)	72 (62–88)	100 (100–100)
Symptoms, median (IQR)	52 (41–68)	96 (88–97)
ADL, median (IQR)	69 (61–89)	100 (100–100)
Sport/Rec., median (IQR)	42 (31–54)	100 (100–100)
PA, median (IQR)	38 (9–44)	100 (100–100)
QOL, median (IQR)	44 (30–56)	100 (100–100)
Clinical entities of groin pain		
Adductor‐related, *n* (%)	20 (100)	—
Pubic‐related, *n* (%)	4 (20)	—
Inguinal‐related, *n* (%)	7 (35)	—
Iliopsoas‐related, *n* (%)	14 (70)	—
Total number of clinical entities, mean (SD)	3 (2)	—
Pubic‐related radiographic findings^c^		
Bone lucency, *n* (%)	13 (65)	—
Proliferation, *n* (%)	12 (60)	—
Fragmentation, *n* (%)	0 (0)	—
Sclerosis, *n* (%)	2 (10)	—
Pubic joint space width, mm (SD)	3 (1)	—
Narrow pubic joint space width, *n* (%)	9 (45)	
Hip‐related radiographic morphologies^c^		
Cam morphology, *n* (%)	5 (25)	—
Pincer morphology, *n* (%)	1 (5)	—
Borderline dysplasia, *n* (%)	6 (30)	—
Dysplasia, *n* (%)	0 (0)	—

*Note:* Definitions: Cam morphology = alpha angle ≥ 60° on a cross‐table lateral radiograph, Pincer morphology = LCEA ≥ 40° OR LCEA ≥ 35° and an acetabular index angle < 0°, borderline hip dysplasia = LCEA 20°–25°, hip dysplasia = LCEA < 20° OR Acetabular index angle > 13°.

Abbreviations: IQR, interquartile range; *n*, number; SD, standard deviation.

^a^ Sport level data was missing for one symptomatic athlete.

^b^ Education level data were missing for two asymptomatic athletes.

^c^ Pubic‐ and hip‐related radiographic parameters as evaluated post‐inclusion by an independent musculoskeletal radiologist.

### 2.4 Pain Sensitivity

Pain sensitivity was assessed with a pressure‐based quantitative sensory testing (QST) protocol. QST is a standardized psychophysical method for assessing peripheral and central nociceptive function [13]. Mechanical pressure was selected as the primary stimulus because it is non‐invasive and closely mimics deep tissue pain arising from muscles, tendons, and bone [14]. In this study, the QST protocol included assessment of (1) pressure pain thresholds [26] with a handheld algometer (1 cm^2^ probe, Somedic, Sweden) and (2) pain detection thresholds [26], pain tolerance thresholds [26], temporal summation of pain [26] and conditioned pain modulation [27] assessed with a computer‐controlled cuff‐algometer (Nocitech and Aalborg University, Denmark) [26, 27]. Together, the QST protocol provides indications of local and widespread mechanical hyperalgesia and the function of pro‐nociceptive (facilitated) and anti‐nociceptive (inhibitory) central pain mechanisms [14].

To standardize pain sensitivity assessments, each leg was designated as either the index‐side or secondary‐side. In symptomatic athletes, the index‐side was the most painful, while the secondary‐side was the least painful or pain‐free side. In asymptomatic athletes, side designation was randomized with no consideration of an index‐ or secondary‐side.

#### 2.4.1 Pressure Pain Thresholds

Pressure pain thresholds were defined as the pressure (kPa) when athletes started to experience pain from applied mechanical pressure. Pressure pain thresholds were used to assess local and widespread mechanical pain sensitivity. Local mechanical hyperalgesia is indicated by low pressure pain thresholds at painful sites, whereas widespread hyperalgesia is indicated by low pressure pain thresholds at remote, non‐painful sites or at multiple painful sites widespread throughout the body [14].

Pressure pain thresholds were assessed bilaterally at the adductor longus muscle belly (ADL), adductor longus muscle origin (ADL Origin), iliopsoas just inferior to the inguinal ligament (Iliopsoas), pubic bone (Pubic Bone), pubic symphysis joint (Pubic Symphysis, not measured bilaterally), gluteus maximus muscle (Glut Max), and contralateral lateral elbow epicondyle (Elbow, not measured bilaterally). The Glut Max and Elbow sites served as reference sites to evaluate widespread hyperalgesia.

Testing always began on the secondary side. The algometer was applied perpendicular to the skin at 30 kPa/s with 1000 kPa as a safety limit [26]. Athletes pressed a button to indicate the transition from pressure to pain [26]. Two pressure pain thresholds were obtained per site, separated by approx. 1 min, and averaged for analysis [26]. Test–retest reliability for pressure pain thresholds is ICC: 0.87–0.89 [26].

#### 2.4.2 Pain Detection and Tolerance Thresholds

Pain detection and pain tolerance thresholds were used to assess widespread mechanical pain sensitivity, as the cuff‐algometer applies pressure to the lower leg using a tourniquet cuff placed 5 cm below the tibial tuberosity [26, 27]. Accordingly, low pain detection and pain tolerance thresholds may indicate widespread hyperalgesia, as the lower leg is anatomically independent of the groin.

Pain detection and pain tolerance thresholds were assessed once per side, starting with the index‐side. The tourniquet cuff was inflated using a ramp increase in cuff‐pressure from 0 to 100 kPa at 1 kPa/s [26]. Athletes continuously rated pain intensity on an electronic visual analogue scale (VAS, 0–10 cm, “no pain” to “worst pain imaginable”), and terminated the test when pain became intolerable or VAS reached 10 [26]. Pain detection threshold was defined as the pressure at which VAS first exceeded 1 cm [26]; pain tolerance threshold was defined as the pressure at termination [26]. Test–retest reliability of pain detection threshold and pain tolerance threshold at the lower leg using a cuff algometer is ICC: 0.74–0.87 [26].

#### 2.4.3 Temporal Summation of Pain

Temporal summation of pain is considered a surrogate measure of central pro‐nociceptive pain mechanisms and increased dorsal horn excitability in response to repeated identical nociceptive stimuli (the wind‐up phenomenon) [26].

Temporal summation of pain was assessed on the index‐side using 10 repeated cuff‐pressure stimulations on the index‐side [26]. Each stimulus involved rapid inflation (100 kPa/s) to the athlete's pain tolerance threshold (a painful stimulus) for 1 s, followed by deflation to 5 kPa (a non‐painful stimuli) for 2 s [26]. Athletes rated pain intensity of each stimulus [26]. Mean VAS scores were calculated for intervals 1–4 (VAS‐1), intervals 5–7 (VAS‐2) and intervals 8–10 (VAS‐3) [26]. Temporal summation of pain was expressed as the ratio between VAS‐3 and VAS‐1. Facilitated TSP is indicated by a TSP‐ratio greater than 2.48 and normal TSP is indicated by a ratio lower than 2.48 [28]. Test–retest reliability of temporal summation of pain using this procedure is ICC: 0.6 [26].

#### 2.4.4 Conditioned Pain Modulation

Conditioned pain modulation is considered a surrogate measure of central anti‐nociceptive pain mechanisms and mechanisms that regulate the descending pain modulation and diffuse noxious inhibitory control [27].

Conditioned pain modulation was assessed using a constant cuff conditioning stimulus applied to the secondary‐side and a simultaneous reassessment of pain detection threshold and pain tolerance threshold (see above) on the index‐side [27]. The conditioning stimulus pressure intensity corresponded to 75% of the pain tolerance threshold on the secondary‐side. The conditioned pain modulation effect was defined as the absolute differences in pain detection and pain tolerance thresholds from baseline to the conditioning phase [27]. Test–retest reliability of conditioned pain modulation using this procedure is ICC: 0.53–0.75 [27].

### 2.5 Psychological Factors

Thirteen brief screening questions captured pain catastrophizing, fear of movement, pain self‐efficacy, depression, anxiety, stress, loneliness, and sleep (see Table 3) [29, 30, 31]. Answers were rated from 0 (not affected) to 10 (highly affected) [30, 31], except for sleep hours and pain self‐efficacy (scored 0–6 and summarized to 0–12, with 0 being the lowest) [29]. The questions demonstrate acceptable concurrent validity versus full questionnaires [29, 30, 31].

### 2.6 Muscle Function

Muscle function was assessed as maximal isometric and explosive strength during hip adduction squeeze and hip abduction‐press testing with a handheld dynamometer recording force (N) at 100 Hz (MicroFET2, Hoggan, Scientific L.L.C., Salt Lake City, USA) [23, 32]. Athletes performed a 50% and a 100% effort warm‐up trial, followed by three 100% trials squeezing/pressing as fast and hard as possible [23]. Maximal isometric strength was reported as normalized peak torque (Nm/kg) calculated from highest force across valid trials, lever arm (from the anterior superior iliac spine to the dynamometer's center of pressure) and body mass [23]. Maximal explosive strength was reported as normalized rate of torque development (RTD, Nm/s/kg) over 0–100 milliseconds (RTD_100_) and 0–200 milliseconds (RTD_200_) [23]. RTD was calculated as the average slope of the torque–time curve within each time interval [22, 23]. Force onset was defined as 6.7 N above baseline force [23]. Signal filtering and gravity correction were not applied [23]. Test–retest reliability of comparable measures using handheld dynamometry has previously been reported by Ishøi et al., with ICCs of 0.93 for peak torque, 0.63 for RTD_100_, and 0.86 for RTD_200_ [23].

Following each valid trial, test‐evoked groin pain intensity was recorded on a 0–10 numeric rating scale (NRS) from “no pain” to “worst pain imaginable” [24, 32].

### 2.7 Descriptive Data

Age, anthropometrics, sport exposure/level, pain duration, mechanism, prior treatment, pain medication use, and education were recorded. Symptomatic athletes rated current, worst, least, and usual groin pain over the past week using a 0–10 NRS. Distribution of usual groin pain during sport was drawn on digital body charts in Navigate Pain (Aglance Solutions, Aalborg Denmark) [4]. Hip‐ and groin‐related disability was assessed with the revised HAGOS [19].

### 2.8 Sample Size

This study was designed as an explorative study with a flat outcome structure and no predefined primary outcome. This was prioritized to identify potentially clinically meaningful between‐group differences across the outcome domains of pain sensitivity, psychological factors, and muscle function to inform future hypotheses and study designs. Accordingly, the study was powered to detect large between‐group effect sizes (Cohen's *d* > 0.8), rather than prioritize statistical significance. Assuming effect sizes of *d* ≥ 0.9, 80% power, and *α* = 0.05, a sample of 20 symptomatic and 20 asymptomatic athletes was required.

### 2.9 Statistics

Continuous data are summarized as mean (SD) or median (IQR); categorical data as *n* (%). Pain sensitivity and muscle function variables were compared between groups using two‐tailed unpaired Welch's *t*‐tests, and effect sizes were calculated as Cohen's *d* with 95% confidence intervals. To control for bilateral pain, the index and secondary sides were compared separately, with the symptomatic athletes sub‐grouped based on uni‐ or bilateral groin pain for the secondary‐side comparisons. To account for repeated measures subject variability in pressure pain threshold, a sensitivity analysis was performed using a two‐way mixed model analysis of variance (ANOVA) with the between subject factor (Group) and within subject factor (Site: adductor longus muscle/origin, iliopsoas, pubic bone, pubic symphysis, gluteus maximus, elbow). For the ANOVA model, Greenhouse–Geisser corrections are reported as sphericity was violated, and Tukey adjustments were applied to the post hoc *p*‐values.

Psychological variables were compared using Wilcoxon rank‐sum tests with effect sizes as rank‐biserial correlation *r*.

Effect sizes were interpreted as small (0.20–0.49), moderate (0.50–0.79) and large (≥ 0.8). Both unadjusted *p*‐values and Benjamini‐Hochberg adjusted *p*‐values are reported. Analyses were performed in RStudio 2022.12.0 + 353 using R version 4.3.0.

## 3 Results

### 3.1 Participants

**FIGURE 1:**
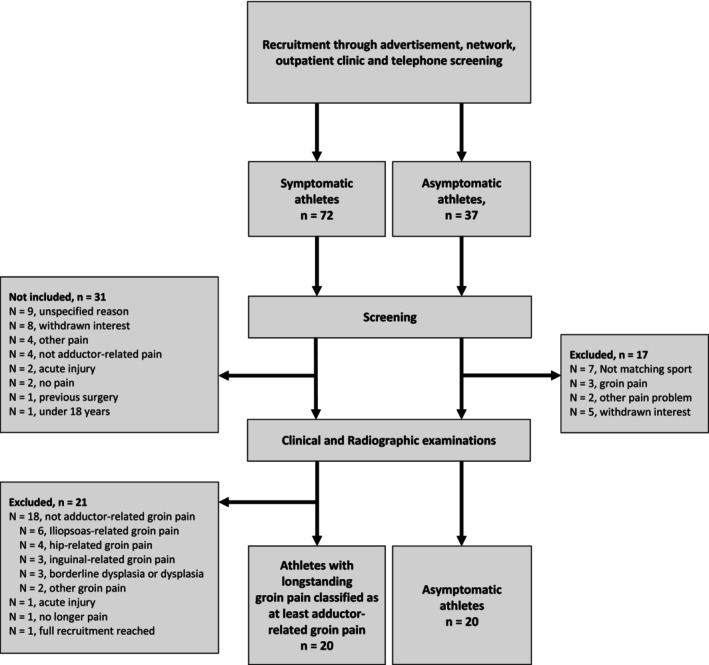
Participant flow.

**FIGURE 2:**
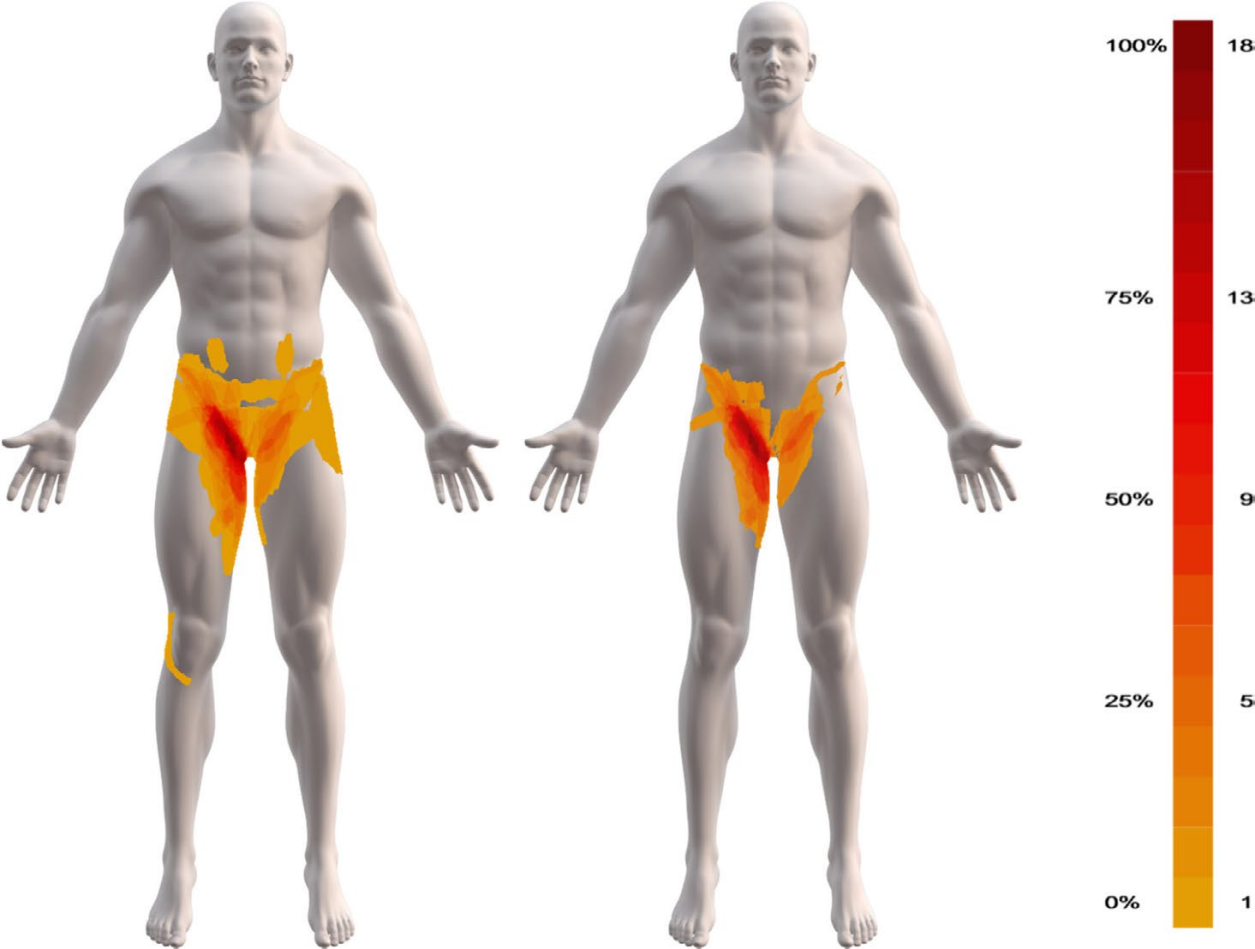
Overlay drawings of usual groin pain during sport activity. Left drawing illustrates all areas drawn by a minimum of one symptomatic athlete. Right drawing illustrates areas drawn by a minimum of two symptomatic athletes. Drawings were “normalized/flipped” according to the most painful side, so the right side of the body chart is the most painful side, and the left side is the least painful side. The color‐scheme range reflects the maximum overlap and the minimum overlapping area, shown in percent and the absolute number of drawings.

Participant flow and characteristics are presented in Figure 1 and Table 1, respectively. All symptomatic athletes presented with adductor‐related groin pain, while iliopsoas‐, inguinal‐, and pubic‐related groin pain were present in 70%, 37%, and 20% of symptomatic athletes, respectively (Tables 1 and A1). Distributions of usual groin pain during sport are visualized in Figure 2. Additional clinical and radiographic findings are available in Tables A1 and A2.

### 3.2 Pain Sensitivity

#### 3.2.1 Pressure Pain Thresholds

**FIGURE 3:**
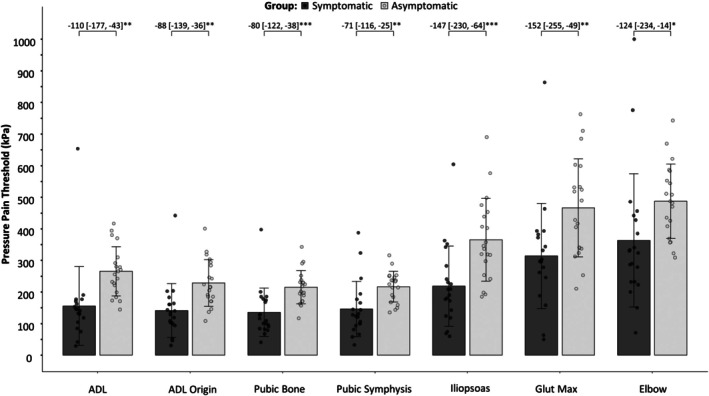
Pressure pain thresholds on the index‐side. Bars are means. Error bars are standard deviations. Dots are individual data points. *p < 0.05, **p < 0.01, ***p < 0.001. Measurement sites: ADL = midpoint of adductor longus muscle belly, ADL Origin = adductor longus origin at the pubic bone, Pubic Bone = pubic bone just adjacent to the symphysis joint, Pubic Symphysis = anterior part of the symphysis joint, Iliopsoas = Iliopsoas tendon just distally to the inguinal ligament, Glut Max = midpoint of the gluteus maximus muscle, Elbow = lateral elbow condyle contralateral to the index side.

**FIGURE 4:**
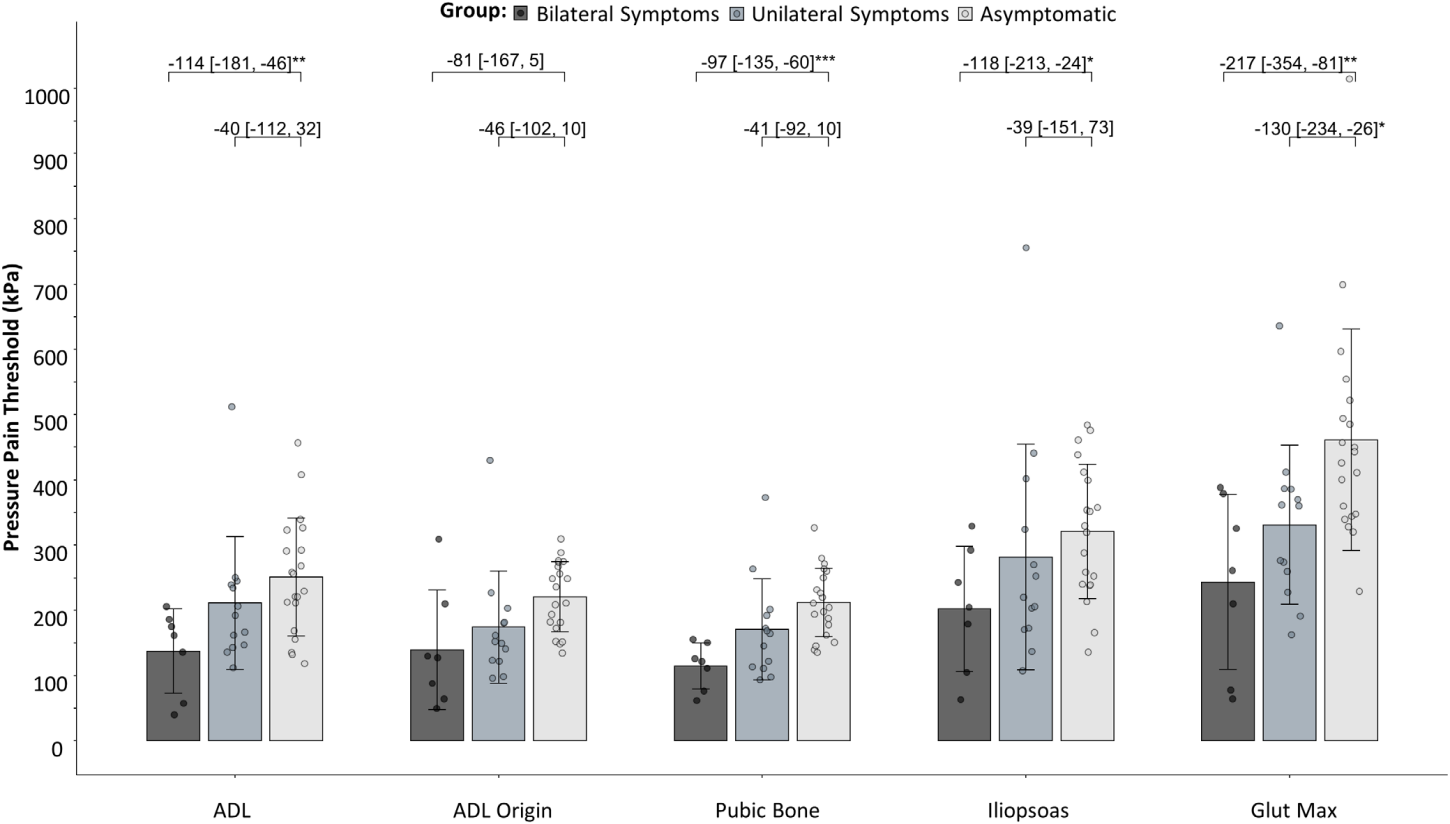
Pressure pain thresholds on the secondary‐side. Bars are means. Error bars are standard deviations. Dots are individual data points. *p < 0.05, **p < 0.01, ***p < 0.001. Measurement sites: ADL = midpoint of adductor longus muscle belly, ADL Origin = adductor longus origin at the pubic bone, Pubic Bone = pubic bone just adjacent to the symphysis joint, Pubic Symphysis = anterior part of the symphysis joint, Iliopsoas = iliopsoas tendon just distally to the inguinal ligament, Glut Max = midpoint of the gluteus maximus muscle, Elbow = lateral elbow condyle contralateral to the index side.

Pressure pain thresholds for the index‐side and secondary‐side are presented in Figures 3 and 4. Site‐specific effect sizes are provided in Table A3. At the group level, symptomatic athletes demonstrated lower pressure pain thresholds than asymptomatic athletes across sites on the index‐side, with moderate to large effect sizes (*d* = 0.7 to 1.2, Figure 3, Table A3). The two‐way ANOVA analyses showed significant main effects of group (*F* (1, 38) = 13.2, *p* = 0.0008, ges = 0.19) and measurement site (*F* (6, 228) = 78.7, *p* < 0.001, ges = 0.42), with no evidence of a group × site interaction (*F* (6, 228) = 1.9, *p* = 0.139). Post hoc contrasts are reported in Tables A5, A6, A7.

On the secondary side, athletes with bilateral groin pain showed lower pressure pain thresholds across most sites, with large effect sizes (*d* > 1.2, Figure 4). In contrast, athletes with unilateral groin pain showed lower pressure pain thresholds across sites, but only with large effects for the Glut Max site (*d* = 0.9, Figure 4).

Across pressure pain threshold sites, the estimated effects were accompanied by substantial uncertainty, as reflected by wide confidence intervals (spanning up to 1.3 points on the index‐side and 2.0 points on the secondary‐side; Table A3). Importantly, individual‐level data showed overlap between groups. Several symptomatic athletes showed pressure pain thresholds comparable to or higher than those measured in asymptomatic athletes across multiple sites (Figures 3 and 4), indicating considerable heterogeneity in pain sensitivity within the athletes with long‐standing groin pain.

#### 3.2.2 Pain Detection Thresholds, Pain Tolerance Thresholds, Temporal Summation of Pain and Conditioned Pain Modulation

Pain detection thresholds, pain tolerance thresholds, temporal summation of pain and conditioned pain modulation measures are presented in Table 2. At the group level, symptomatic athletes showed lower pain detection thresholds on the index‐side, with moderate effect size and very wide confidence intervals (*d* = 0.7, 95% CI: 0.07–1.35). Similarly, athletes with unilateral groin pain also showed lower pain detection thresholds on the secondary side, with large effect size but very wide confidence intervals (*d* = 1.0, 95% CI: 0.27–1.7).

**TABLE 2:** Pain detection threshold, pain tolerance threshold, temporal summation of pain, and conditioned pain modulation.

	Symptomatic	Asymptomatic	Symptomatic vs Asymptomatic				ES	95% CI
	Mean (SD)	Mean (SD)	Diff	95% CI	*p*	Adj. *p*		
**Index side**								
Pain detection threshold (kPa)	20 (8)	26 (11)	−7	−13 to −1	0.03	0.09	0.7	0.1 to 1.4
Pain tolerance threshold (kPa)	89 (15)	89 (15)	0	−9 to 10	0.98	0.98	0.0	−0.6 to 0.6
Pain intensity at pain tolerance threshold (cm)	9.7 (0.5)	8.9 (1.3)	0.8	0.2 to 1.4	0.02	0.06	0.8	0.2 to 1.5
Conditioned pain detection threshold (kPa)	30 (14)	38 (19)	−8	−19 to 3	0.13	0.29	0.5	−0.1 to 1.1
Conditioned pain tolerance threshold (kPa)	89 (23)	92 (12)	−3	−15 to 9	0.62	0.76	0.2	−0.5 to 0.8
Pain intensity at conditioned pain tolerance threshold (cm)	9.3 (0.8)	8.1 (2.0)	1.2	0.2 to 2.2	0.02	0.06	0.8	0.1 to 1.5
Temporal summation pain ratio	1.5 (0.7)	1.6 (0.7)	−0.1	−0.5 to 0.4	0.68	0.76	0.1	−0.5 to 0.8
**Conditioned pain modulation effect**								
Δ Pain detection threshold (kPa)	10 (12)	12 (14)	−2	−10 to 7	0.66	0.76	0.1	−0.5 to 0.8
Δ Pain tolerance threshold (kPa)	0 (20)	3 (5)	−3	−13 to 7	0.53	0.76	0.2	−0.4 to 0.8
**Secondary side**								
**Unilateral pain, *n* = 13**								
Pain detection threshold (kPa)	20 (7)	29 (10)	−9	−15 to −3	0.01	0.04	1.0	0.3 to 1.7
Pain tolerance threshold (kPa)	90 (13)	90 (14)	0	−10 to 10	0.94	0.98	0.0	−0.7 to 0.7
Pain intensity at pain tolerance threshold (cm)	9.8 (0.3)	8.8 (1.3)	1.0	0.4 to 1.7	0.004	0.04	1.0	0.3 to 1.7
**Bilateral pain, *n* = 7**								
Pain detection threshold (kPa)	23 (10)	29 (10)	−6	−16 to 4	0.18	0.37	0.6	−0.3 to 1.5
Pain tolerance threshold (kPa)	83 (14)	90 (14)	−7	−20 to 7	0.29	0.53	0.5	−0.4 to 1.4
Pain Intensity at pain tolerance threshold (cm)	9.8 (0.3)	8.8 (1.3)	1.0	0.3 to 1.7	0.005	0.04	1.0	0.3 to 1.7

Abbreviations: Adj. *p*, Benjamini‐Hochberg adjusted *p*‐values; cm, centimeters; ES, effect size estimated as Cohen's *d*; kPa, kilopascal; SD, standard deviation; ΔPDT, Absolute change in PDT; ΔPTT, absolute change in PTT.

No consistent between‐group differences were observed for temporal summation of pain or conditioned pain modulation effect. During pain detection and tolerance thresholds assessments, the 100 kPa pressure limit was reached by 19 and 17 athletes on the index‐side and secondary‐side, respectively. The conditioning stimulus during the CPM protocol reached an average intensity of VAS = 7.1 across all athletes.

### 3.3 Psychological Factors

Psychological factor measures are presented in Table 3. Symptomatic athletes reported less pain self‐efficacy (*r* = 0.6) and higher levels of pain catastrophizing (*r* = 0.6), fear of movement (*r* = 0.4–0.5), and depressive symptoms (*r* = 0.3–0.4), although no large effect sizes were observed. No between‐group differences were observed for the remaining psychological factors.

**TABLE 3:** Psychological factors.

Psychological factors	Symptomatic athletes		Asymptomatic athletes		*p*	Adj. *p*	ES
	Median	IQR	Median	IQR			
**Pain catastrophizing**							
Context: How often do you have had the following thoughts about your pain during the past 4 weeks?							
Q1: “When I feel the pain, it is terrible, and I feel that it's never going to get better” Response: 0 “never do that” to 10 = “always do that”	4	1 to 6	0	0 to 0	0.0002	0.0008	0.6
Q2: “When I feel pain, I feel that I can't stand it anymore” Response: 0 “never do that” to 10 = “always do that”	1	0 to 3	0	0 to 0	0.0004	0.001	0.6
**Fear of movement**							
Context: To what extent do you disagree or agree with the statements?							
Q1: “Physical activity might damage me” Response: 0 = “completely disagree” to 10 = “completely agree”	3	2 to 7	1	0 to 2	0.01	0.02	0.4
Q2: “I should not do physical activities which (might) make my pain worse” Response: 0 = “completely disagree” to 10 = “completely agree”	6	6 to 7	1	0 to 2	0.0004	0.01	0.5
**Pain self‐efficacy**							
Context: Please rate how confident you are that you can do the following things at present, despite the pain							
Q1: “I can do some form of work, despite the pain (“work” includes housework and paid and unpaid work)” Response: 0 = not at all confident to 6 = completely confident	6	6 to 6	6	6 to 6	0.02	0.03	0.4
Q2: “I can live a normal lifestyle, despite the pain” Response: 0 = not at all confident to 6 = completely confident	5	5 to 6	6	6 to 6	0.00006	0.0006	0.6
Total score	11	10 to 12	12	12 to 12	0.00006	0.0006	0.6
**Depression**							
Q1: “During the past month, have you often felt sad, depressed or had a sense of hopelessness?” Response: 0 = “NEVER” to 10 = “all the time”	3	1 to 5	1.0	1 to 2	0.04	0.06	0.3
Q2: “During the past month, have you felt bothered by little interest or pleasure in to do something?” Response: 0 = “never” to 10 = “all the time”	3	1 to 3	1.0	0 to 1	0.02	0.03	0.4
**Anxiety**							
Q: “Do you feel anxious?” Response: 0 = “not at all anxious” to 10 = “quite anxious”	1	0 to 3	1.0	0 to 2	0.98	0.98	0.0
**Stress**							
Q: “Do you feel stressed?” Response: 0 = “not at all stressed” to 10 = “very stressed”	2	0 to 2	3	2 to 4	0.10	0.12	0.3
**Loneliness**							
Q: “Are you lonely?” Response: 0 “not at all lonely” to 10 = “quite lonely”	1	0 to 2	1	0 to 1	0.59	0.62	0.1
**Sleep**							
Q1: How would you rate the average quality of your sleep over the past week? Response: 0 = “best quality” to 10 = “worst quality”	5	2 to 6	3	2 to 5	0.55	0.62	0.1
Q2: How many hours did you sleep on average per night over the past week? Response: hours	7	6 to 7	7	7 to 8	0.14	0.17	0.2

Abbreviations: ES, effect size estimated as rank‐Biserial correlation; IQR, interquartile range; Q1, Question 1; Q2, Question 2.

### 3.4 Muscle Function

**FIGURE 5:**
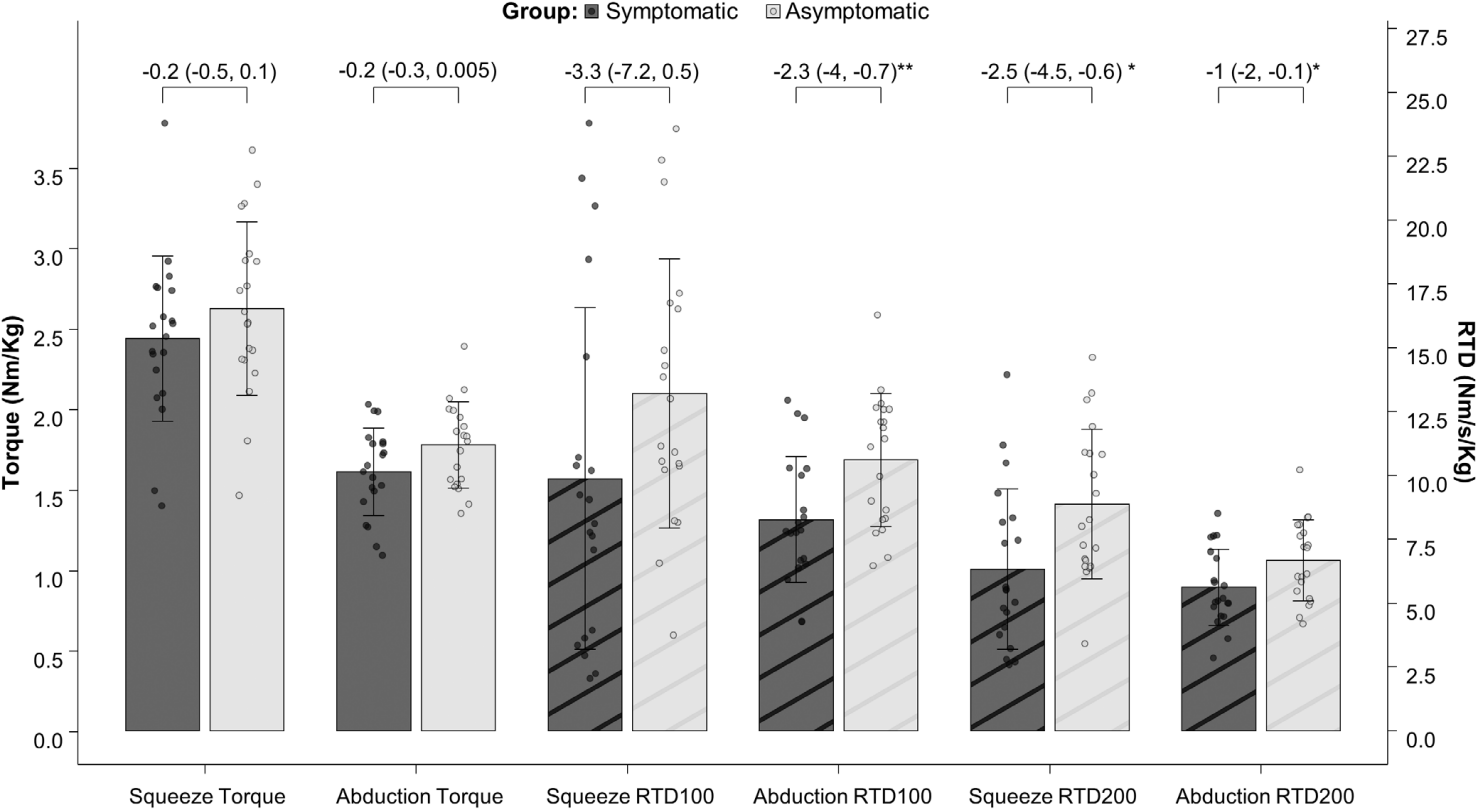
Muscle function. Bars are means. Error bars are standard deviations. Dots are individual data points. Full color bars are maximal isometric strength. Pattern bars are explosive strength. Kg, kilogram, Nm, newton meter, RTD, rate of torque development, s, seconds, *p < 0.05, **p < 0.01.

Maximal and explosive strength measures are presented in Figure 5 and Table A4. At the group level, symptomatic athletes showed lower explosive strength with moderate to large effect sizes but wide confidence intervals for hip adduction RTD_200_ (*d* = 0.8, 95% CI: 0.2 to 1.5), hip abduction RTD_100_ (*d* = 0.9, 95% CI: 0.3 to 1.6), and hip abduction RTD_200_ (*d* = 0.7, 95% CI: 0.0 to 1.3). Importantly, individual data displayed notable overlap between groups, indicating substantial heterogeneity in muscle function among symptomatic athletes (Figure 5).

Symptomatic athletes reported severe (NRS median: 7, IQR: 4–8) and mild (NRS median: 3, IQR: 1–5) groin pain from hip adduction and abduction testing, respectively. Asymptomatic athletes reported no provoked pain.

## 4 Discussion

By integrating new neurophysiological, psychological, and biomechanical measures, this study aimed to identify factors in male athletes with long‐standing groin pain that may be relevant for informing a more individualized biopsychosocial management strategy to improve pain, disability, and return to sport [11, 12]. This approach contrasts with prevailing biomedical models, which primarily focus on painful anatomical structures and physical deficits [7, 9]. Accordingly, we compared measures of pain sensitivity, psychological factors, and muscle function (maximal and explosive strength) between male athletes with long‐standing groin pain and matched asymptomatic controls.

At the group level, symptomatic athletes showed altered pain sensitivity (widespread mechanical hyperalgesia), greater pain catastrophizing, fear of movement and depressive symptoms, lower pain self‐efficacy, and lower explosive hip adduction and abduction strength. However, individual‐level data often overlapped between groups, highlighting substantial heterogeneity among athletes with long‐standing groin pain.

Taken together, these findings suggest that long‐standing groin pain may range from a relatively localized musculoskeletal issue driven predominantly by nociceptive pain to more complex presentations that may be driven by nociplastic pain mechanisms with alterations in systemic neurophysiological and psychological processes. In the latter and more complex presentations, clinicians may need to adopt a broader biopsychosocial assessment and treatment approach.

### 4.1 Pain Sensitivity

Moderate to large between‐group differences were observed in pressure pain thresholds, with lower values in the symptomatic athletes across multiple sites on both the index‐side, the secondary‐side, and control sites at the gluteus maximus and elbow. However, individual‐level data revealed substantial overlap between groups, with several symptomatic athletes showing pressure pain thresholds corresponding to those of asymptomatic athletes. A similar pattern was observed for pain detection thresholds, whereas pain tolerance threshold, temporal summation of pain, and conditioned pain modulation did not differ between groups. Collectively, these findings suggest that a large proportion of male athletes with long‐standing groin pain may show widespread mechanical hyperalgesia, and no clear evidence of altered pain modulation under the present testing conditions.

The pattern of widespread mechanical hyperalgesia aligns with mechanistic interpretations suggesting that symptom severity reflects sensitization across peripheral and central pathways [13, 14]. This demonstration of widespread hyperalgesia, in the absence of clear alterations in pain modulation, extends prior evidence from a study in male athletes with groin pain, which reported local mechanical hyperalgesia at the adductor longus tendon regardless of the clinical entity of groin pain, but found no evidence of widespread mechanical hyperalgesia [15]. Two main points may partly explain these discrepancies. First, pain severity may serve as a proxy for the extent of altered nociceptive function [13, 14]. Symptomatic athletes in the present study reported long symptom duration (median 52 weeks), severe pain intensity (7 on the NRS), and high disability (low HAGOS scores, Table 1), whereas pain severity was not reported in the previous study [15]. Second, long‐standing groin pain often presents as multiple coexisting groin pain entities [4, 6] and may involve compensatory loading strategies of several muscles surrounding the pelvis. This aligns with the concept of long‐standing groin pain as a pelvic overload syndrome, in which multiple structures are overloaded and stressed simultaneously rather than a single isolated tissue [33, 34]. When and if several structures generate nociceptive stimuli concurrently, the summation of input may facilitate nociception and create a vicious cycle of sensitization in the peripheral groin structures and the central T12–L5 spinal segments, thereby creating and maintaining pain [13, 14].

However, these explanations do not readily account for the lower pressure pain thresholds at the elbow, which instead suggests a degree of more generalized hypersensitivity likely driven sensitization independent of the hip and groin at the C5‐T1 segments or supraspinal and cortical areas [13, 14]. Interestingly, this generalized hypersensitivity contrasts with the absence of group differences in temporal summation of pain and conditioned pain modulation. Several non‐mutually exclusive explanations may account for this apparent discrepancy. Firstly, both applied temporal summation of pain and conditioned pain modulation paradigms show only moderate test–retest reliability and have an upper stimuli limit of 100 kPa [26, 27], which may limit the ability to detect group differences. Secondly, temporal summation of pain and conditioned pain modulation may reflect partially distinct neurophysiological mechanisms [13, 14]; temporal summation of pain primarily captures facilitatory spinal processes (e.g., wind‐up) [26], whereas conditioned pain modulation reflects the net balance of descending inhibitory and facilitatory control [27]. It is therefore plausible that symptomatic athletes exhibited a change in mechanical pain sensitivity without impairments in dynamic pain modulation. Thirdly, regular physical activity and athletic training may preserve or enhance endogenous pain inhibitory capacity, potentially explaining intact conditioned pain modulation in athletes with long‐standing groin pain despite heightened pain sensitivity [35].

The present findings contrast with patterns observed in athletes with lower‐limb tendinopathies, who typically show local hyperalgesia rather than widespread hyperalgesia [36], and normal conditioned pain modulation, when assessed using other testing paradigms [37]. In contrast, widespread hyperalgesia, impaired conditioned pain modulation, and facilitated temporal summation of pain have been observed in severe pain conditions like patellofemoral pain, low back pain, fibromyalgia, whiplash, knee osteoarthritis, and chronic widespread pain [13, 14]. Furthermore, facilitated temporal summation of pain has been reported in 41% (23 of 51) of patients with hip‐related groin pain when assessed with Von Frey filaments on the finger [16]. The divergence from our temporal summation of pain findings may reflect differences in stimulus modality and anatomical site, as von Frey filaments preferentially activate cutaneous nociceptors, whereas cuff‐algometry at the lower leg targets deep‐tissue nociceptors [26]. Another explanation could be differences in symptom severity and source of nociceptive. Patients with hip‐related groin pain reported greater resting pain intensity (NRS 4) and substantially longer symptom duration (mean 31 months) than the athletes in the present study. This supports the notion that higher pain severity may be related to more extensive nociceptive alterations. Additionally, the rich mechanoreceptor and nociceptors innervation of the hip joint may represent a more potent and continuous driver of altered nociceptive function than the adductor muscle‐tendon‐bone complex. It is not unreasonable to think that habitual hip‐joint loading could more extensively sensitize central pain pathways.

### 4.2 Psychological Factors

At the group level, male athletes with long‐standing groin pain reported higher pain catastrophizing and fear of movement, more depressive symptoms, and lower pain self‐efficacy compared with the asymptomatic athletes. However, the between‐group differences were small to moderate, and individual‐level data revealed normal psychological profiles of several symptomatic athletes. Consistent with this heterogeneity, both median values and absolute individual scores among symptomatic athletes were generally below those reported in patients with severe long‐standing pain conditions [31].

In patients with severe long‐standing pain conditions, the recommended thresholds for screening psychological factors include an average pain‐catastrophizing score ≥ 7 (or single‐item score ≥ 7); fear‐of‐movement average ≥ 7 (or single‐item ≥ 5); depressive‐symptom score ≥ 7 or ≥ 6 on both questions; and pain self‐efficacy total < 8 indicating low pain self‐efficacy. When these thresholds are applied to the present cohort, most symptomatic athletes do not meet criteria indicative of adverse psychological factors. This suggests that although adverse psychological states or traits may be present, they typically do not reach magnitudes observed in patients with severe long‐standing pain conditions [31]. Importantly, this pattern should not be interpreted as a lack of relevance of psychological factors, but rather as a reflection of sports‐specific pain contexts and coping demands.

Supporting this interpretation, athletes with long‐standing pubic and/or adductor‐related groin pain have previously shown mean scores of 22 (range: 5–42) on the full Pain Catastrophizing Scale (PCS; range 0–52), with improvements observed during and following rehabilitation [10]. These baseline values exceed reference values from healthy individuals (PCS ≈ 15) [38], and patients with lower‐limb pain (PCS ≈ 13) [38], although not reaching the clinically relevant threshold (PCS ≥ 30) from patients with long‐standing pain [31].

Collectively, these findings support the importance of screening for and further exploring psychological factors in male athletes with long‐standing groin pain. It is not unreasonable to think that maladaptive fear of movement, pain catastrophizing and low pain self‐efficacy may be context‐specific, emerging particularly during high‐load or high‐risk sport situations that may evoke expectations of pain such as maximal sprinting, rapid change of direction or kicking [39].

### 4.3 Muscle Function

At the group‐level, small to large differences were observed in hip muscle function, with lower explosive strength in male athletes with long‐standing groin pain. However, lower muscle function was not uniform with individual‐level data showing multiple symptomatic athletes achieving strength measures comparable to asymptomatic athletes (Figure 5). The significant differences in adduction RTD_200_ (33%), abduction RTD_200_ (17%), and abduction RTD_100_ (24%) clearly exceeded the corresponding minimal detectable changes (MDC) of 8%, 7%, and 11%, respectively [23]. Similarly, the non‐significant differences in adduction squeeze RTD_100_ (29%), adduction peak torque (7%), and abduction peak torque (10%) also exceeded MDCs of 16%, 5%, and 3%, respectively [23]. These effect sizes indicate that despite the individual‐level heterogeneity, the differences across strength measures were not caused by measurement errors.

Explosive hip strength has not previously been measured in athletes with long‐standing groin pain, although deficits in explosive hip flexion and extension have been reported in patients with hip‐related groin pain before [40] and after hip arthroscopy [41]. The present findings for isometric strength align with previous studies using handheld dynamometry that found no significant between‐group differences [21], whereas studies using sphygmomanometers have reported more consistent deficits [21]. Collectively, these studies suggest that explosive strength may be more sensitive to the presence of long‐standing groin pain than peak torque capacity [22], as also seen in populations with upper limb/shoulder pain [42].

A possible interpretation is that lower explosive strength reflects acute pain‐related neural inhibition rather than structural muscle weakness [42]. Explosive testing with rapid, high‐intensity contractions generates high peak biomechanical stresses [22], which may exceed nociceptive thresholds in sensitized groin tissues and trigger an intense barrage of nociceptive stimuli [13, 14]. This nociceptive input may transiently inhibit motor output via spinal and supraspinal mechanisms, disproportionately constraining explosive strength [42]. In contrast, slower maximal contractions produce lower biomechanical stresses, potentially limiting nociceptive stimuli summation and intensity [13, 14], enabling greater motor unit recruitment in painful muscles and compensatory activation of synergist muscles [42], and allowing near‐normal peak torque despite acute provoked groin pain.

Pain‐related cortical inhibition associated with fear of movement and pain catastrophizing may further amplify these effects, particularly during the early phase of explosive contractions as demonstrated and suggested by Sundstrup et al. [42]. This interpretation is consistent with the symptomatic athletes demonstrating higher fear of movement and pain catastrophizing, and underscores the potential interaction between pain sensitivity, psychological factors and muscle function [42].

Taken together, these findings suggest that in many male athletes with long‐standing groin pain, explosive hip strength may be preferentially limited by pain‐related neural mechanisms rather than by loss of maximal strength capacity. This pattern is consistent with altered nociceptive function and motor processes at spinal and supraspinal levels, while emphasizing that neuromuscular impairment in male athletes with long‐standing groin pain exists along a spectrum rather than as a uniform characteristic.

### 4.4 Strengths and Limitations

This case–control study was designed as an exploratory investigation to detect large and potentially clinically relevant signals across neurophysiological, psychological, and muscle function variables that have not previously been comprehensively assessed in male athletes with long‐standing groin pain. A key strength was the integration of standardized quantitative sensory testing, psychological screening, and assessment of both maximal and explosive muscle function in a single case–control study. Additional strengths included matching symptomatic and asymptomatic athletes by sex, age, and sport to reduce potential confounding. The standardized classification of groin pain according to the Doha consensus [5] provided a clear clinical description of the included symptomatic athletes (Table 1 and Table A1), which is highly important due to the heterogeneous clinical presentation with multiple coexisting clinical entities of groin pain.

Several limitations should be acknowledged. The cross‐sectional design limited causal inference, and the explorative analytical approach increases susceptibility to both type I and type II error. The modest sample size (*n* = 40), combined with multiple comparisons and substantial inter‐individual variability, may have limited statistical power to detect small and moderate but potentially relevant effect sizes. To facilitate transparency, both unadjusted and Benjamini‐Hochberg adjusted *p*‐values were reported.

Radiographic measurements performed by the orthopedic surgeon were used for inclusion and classification of groin pain in symptomatic athletes, whereas the radiologist performed measurements were used exclusively for descriptive reporting in Table 1 and Table A2. Consequently, known inter‐rater variability [43, 44] may have influenced both athlete eligibility and descriptive data, contributing to discrepancies in the classification of borderline hip dysplasia based on the lateral center edge angle, which has a minimal detectable change of 8° [43, 44].

Although all included symptomatic athletes had adductor‐related groin pain (as required for study inclusion), the symptomatic athletes were clinically heterogeneous, with an average of two additional groin pain entities (Table 1, Table A1). This coexistence may have influenced the observed differences, particularly in pressure pain thresholds, and limits attribution of findings solely to adductor‐related groin pain. However, such multiple coexisting entities are common and consistent with previous studies of athletes with long‐standing adductor‐related groin pain [6, 45].

Logistical constraints prevented assessor blinding for the experimental session, which may have introduced expectation bias and increased between‐group differences in the assessor‐dependent measures, such as pressure pain thresholds. However, all other measurements, including cuff‐algometry, strength testing, and psychological screening questions, were largely assessor‐independent, limiting the overall impact of bias from the unblinded assessor.

During cuff‐algometry, a substantial proportion of participants reached the upper pressure limit of the device (100 kPa; 19 for the index and 17 for the secondary side), indicating a ceiling effect. This may have reduced the ability to detect between‐group differences in pain tolerance threshold, temporal summation of pain, and conditioned pain modulation.

The brief psychological screening questions were selected to minimize responder burden and enhance feasibility, although they have not undergone psychometric investigation in athletes [30, 31].

A handheld dynamometer with a sampling frequency of 100 Hz was used to assess RTD. This relatively low sampling frequency, along with potential device instability, may increase measurement error and impair precise identification of contraction onset. These factors may particularly affect the validity of the RTD_100_ measures.

Finally, the findings may not be generalized beyond adult male athletes with long‐standing groin pain.

## 5 Conclusion

At the group level, male athletes with long‐standing groin pain showed altered pain sensitivity, elevated levels in adverse psychological factors, and reduced muscle function compared with sex‐, age‐, and sports‐matched asymptomatic athletes.

## 6 Perspective

Long‐standing groin pain is often diagnosed and managed based on a biomedical model that emphasize localized tissue pathology and mechanical tissue overload as the main pain drivers [7, 9]. Accordingly, most contemporary management approaches incorporate pain‐contingent strategies, where exercises and activities are adjusted according to acute pain response during exercises and the implicit assumption that pain primarily reflects tissue damage [8]. While this approach is effective for most athletes (50%–86%) [7, 9], disability is still common after returning to sport [3, 10, 34], and up to 50% do not return to sport at all [7, 9].

The current study highlights that not all athletes with long‐standing groin pain can be adequately characterized by a predominantly nociceptive pain mechanism, as a substantial proportion have features of nociplastic pain [11, 12]. In these athletes with more complex presentation, their exercise, activity, and load tolerance are likely modified and clouded by tissue sensitization as well as complex spinal and supraspinal adaptations. Accordingly, pain‐contingent management strategies such as completely avoiding painful activities and exercises, or insisting on pain reduction before progression may not be an optimal rehabilitation approach [11, 12]. Focusing on pain during treatment as a suggestive marker of tissue damage may inadvertently reinforce pain through hypersensitivity, psychological factors, and reduced muscle function [11, 12]. Thus, a broader biopsychosocial perspective may be required, in which pain responses are contextualized within altered pain processing and negative pain beliefs rather than treated exclusively as signals of tissue damage [11, 12]. Future studies should explore whether more athletes with long‐standing adductor‐related groin pain may achieve a full return to sport with normalized function and disability if pain education [12], graded activities, psychological support, and targeted explosive strength training are integrated alongside current evidence‐based management [8].

## Author Contributions

Conceptualization: M.F.N., L.I., P.H., and K.T.; methodology: M.F.N., L.I. M.B.C., C.J., S.B., T.G.‐N., P.H. and K.T.; formal analysis: M.F.N.; investigation: M.F.N., P.H. and K.T.; resources: M.B.C., S.B., T.G.‐N., P.H., and K.T.; data curation: M.F.N., S.B.; writing – original draft: M.F.N. and K.T.; writing – review and editing manuscript: M.F.N., L.I., M.B.C., C.J., S.B., T.G.‐N., P.H. and K.T.; visualization: M.F.N., S.B.; supervision: P.H., L.I., and K.T.; project administration: M.F.N., P.H., and K.T.; funding acquisition: M.F.N., P.H., L.I., and K.T.

## Funding

This project has received the following grant support from Danske Fysioterapeuters Fond for Forskning, Uddannelse og Praksisudvikling Hovedområde Forskning; Dansk Selskab for Sports Fysioterapi (DSSF) forskningsfond; Hvidovre Hospitals Forskningspulje for Professionsbachelorer 2021; a Danish fund that wishes to be anonymous, Aase og Ejnar Danielsens Fond, The Spies Foundation. TGN is a part of Center for Neuroplasticity and Pain (CNAP) supported by the Danish National Research Foundation (DNRF121), and he receives funding from the Lundbeck Foundation (R441‐2023‐232).

## Ethics Statement

The study protocol was approved by the local ethics committee of the Capital Region in Denmark (H‐20055130).

## Consent

Participants provided written informed consent before undergoing any study related activities. The study was approved by the Danish Capital Region ethics committee (H‐20055130).

## Conflicts of Interest

The authors declare no conflicts of interest.

## Supporting information


**Table S1:** Predetermined order of procedures in the experimental session.

## Data Availability

The data that support the findings of this study are available on request from the corresponding author. The data are not publicly available due to privacy or ethical restrictions.

## Appendix A

**TABLE A1:** Descriptive data from clinical examination of included symptomatic athletes.

Clinical examination	Individual	Right	Left
Palpation			
Adductor longus insertion point at the pubic bone, *n* (%)	20 (100)	16 (80)	8 (40)
Upper insertion of adductor Magnus, *n* (%)	6 (30)	5 (25)	3 (15)
Pubic bone just medial and inferior to the pubic tubercle, *n* (%)	3 (15)	2 (10)	1 (5)
Rectus abdominus insertion/Pyramidalis‐anterior pubic ligament adductor longus complex, *n* (%)	2 (11)	1 (5)	1 (5)
Attachment of the Conjoined tendon, *n* (%)	8 (40)	6 (30)	3 (15)
The superficial inguinal ring, *n* (%)	6 (46)	4 (20)	2 (10)
Psoas muscle belly superior to inguinal ligament, *n* (%)	12 (60)	9 (45)	7 (35)
Iliopsoas muscle tendon inferior to inguinal ligament and the insertion of iliopsoas at the lesser trochanter of the femur, *n* (%)	11 (55)	8 (40)	5 (25)
Pubic symphysis and adjacent pubic bone, *n* (%)	2 (10)		
Resistance, stretch and passive movement tests			
Bilateral adduction squeeze with straight legs, *n* (%)	20 (100)	16 (80)	8 (40)
Passive stretching of the adductor muscles, *n* (%)	8 (40)	6 (30)	3 (15)
Resisted straight and oblique sit‐ups, *n* (%)	5 (25)	4 (20)	2 (10)
Resisted hip flexion in supine, *n* (%)	5 (25)	3 (15)	3 (15)
The modified Thomas test, *n* (%)	6 (30)	4 (20)	2 (10)
Flexion‐Adduction‐Internal‐Rotation (FADIR), *n* (%)	0 (0)	0 (0)	0 (0)
Flexion‐Abduction‐External‐Rotation (FABER), *n* (%)	4 (20)	2 (10)	2 (10)
Diagnostic ultrasound guided intra‐articular anesthetic injection			
Post‐injection palpation of distal iliopsoas, *n* (%)	—	—	—
Post‐injection FADIR‐Test, *n* (%)	—	—	—
Clinical entities of groin pain			
Adductor‐related, *n* (%)	20 (100)	16 (80)	7 (35)
Pubic‐related, *n* (%)	4 (20)	3 (15)	2 (10)
Inguinal‐related, *n* (%)	7 (35)	5 (26)	3 (16)
Iliopsoas‐related, *n* (%)	14 (70)	11 (55)	7 (35)
Cumulative scores			
Total number of positive pain provocation tests, mean (SD)	6 (2)	7 (4)	
Total number of clinical entities, mean (SD)	3 (2)	3 (2)	

Abbreviations: *n*, number, SD, standard deviation.

**TABLE A2:** Descriptive data from radiographic examination of included symptomatic athletes.

Radiographic examinations	Individual	Right	Left
Hip‐related radiographic parameters^a^			
Alpha angle, mean (SD)	53 (11)	52 (9)	56 (13)
Lateral center edge angle, mean (SD)	30 (5)	30 (6)	30 (4)
Acetabular index angle, mean (SD)	6 (4)	6 (4)	5 (4)
Hip joint space width, millimeters, mean (SD)	5 (1)	5 (1)	5 (1)
Cross over sign, *n* (%)	13 (65)	11 (55)	10 (50)
Ischial spine sign, *n* (%)	14 (70)	10 (50)	12 (60)
Posterior wall sign, *n* (%)	7 (35)	6 (30)	5 (25)
Hip‐related radiographic morphologies^a^			
Cam morphology, *n* (%)	5 (25)	2 (10)	4 (20)
Pincer morphology, *n* (%)	1 (5)	0 (0)	1 (5)
Borderline dysplasia, *n* (%)	6 (30)	5 (25)	3 (15)
Dysplasia, *n* (%)	0 (0)	0 (0)	0 (0)
Pubic‐related radiographic parameters^a^			
Bone lucency, *n* (%)	13 (65)	11 (55)	12 (60)
Erosion like configuration (ELC), *n* (%)	13 (65)	11 (55)	12 (60)
ELC central/superior, *n* (%)	10 (50)	9 (45)	8 (40)
ELC inferior, *n* (%)	11 (55)	7 (35)	10 (50)
Cysts, *n* (%)	0 (0)	0 (0)	0 (0)
Proliferation, *n* (%)	12 (60)	10 (50)	10 (50)
Superior proliferation, *n* (%)	5 (25)	4 (20)	4 (20)
Central proliferation, *n* (%)	5 (25)	4 (20)	4 (20)
Inferior proliferation, *n* (%)	11 (55)	8 (40)	8 (40)
Fragmentation, *n* (%)	0 (0)	0 (0)	0 (0)
Central/Superior fragmentation, *n* (%)	0 (0)	0 (0)	0 (0)
Inferior fragmentation, *n* (%)	0 (0)	0 (0)	0 (0)
Sclerosis, *n* (%)	2 (10)	2 (10)	2 (10)
Pubic joint space width, millimeters, mean (SD)	3 (1)	—	—
Narrow pubic joint space width, *n* (%)	9 (45)	—	—
Total number of pubic‐related findings, mean (SD)	5 (3)	5 (4)	

Abbreviations: *n*, number, SD, standard deviation.

^a^ Radiographic parameters were assessed by an independent musculoskeletal radiologist after full inclusion and experimental testing of symptomatic athletes.

**TABLE A3:** Pressure pain thresholds.

	Symptomatic	Asymptomatic	Symptomatic vs. Asymptomatic					
	Mean (SD)	Mean (SD)	Diff	95% CI	*p*	Adj. *p*	ES	95% CI
Index side								
ADL	156 (125)	265 (78)	−110	−177 to −43	0.002	0.007	1.1	0.4 to 1.7
ADL Origin	141 (86)	229 (74)	−88	−139 to −36	0.001	0.006	1.1	0.4 to 1.8
Iliopsoas	218 (127)	365 (131)	−147	−230 to −64	0.0009	0.005	1.1	0.5 to 1.8
Pubic Bone	136 (77)	215 (53)	−80	−122 to −38	0.0005	0.004	1.2	0.5 to 1.9
Pubic symphysis	146 (87)	217 (49)	−71	−116 to −25	0.004	0.009	1.0	0.3 to 1.7
Glut Max	314 (167)	466 (155)	−152	−255 to −49	0.005	0.009	0.9	0.3 to 1.6
Elbow	363 (211)	487 (118)	−124	−234 to −14	0.03	0.04	0.7	0.1 to 1.4
Secondary side								
Unilateral pain, *n* = 13								
ADL	211 (102)	251 (91)	−40	−112 to 32	0.26	0.28	0.4	−0.3 to 1.1
ADL Origin	174 (86)	221 (54)	−46	−102 to 10	0.10	0.12	0.7	−0.04 to 1.4
Iliopsoas	282 (173)	321 (103)	−39	−151 to 73	0.47	0.47	0.3	−0.4 to 1.0
Pubic Bone	171 (78)	212 (53)	−41	−92 to 10	0.11	0.12	0.7	−0.1 to 1.4
Glut Max	331 (122)	461 (170)	−130	−234 to −26	0.02	0.03	0.9	0.1 to 1.6
Bilateral pain, *n* = 7								
ADL	137 (64)	251 (91)	−114	−181 to −46	0.003	0.03	1.3	0.4 to 2.3
ADL Origin	140 (92)	221 (54)	−81	−167 to 5	0.06	0.08	1.2	0.3 to 2.2
Iliopsoas	202 (96)	321 (103)	−118	−213 to −24	0.02	0.03	1.2	0.2 to 2.1
Pubic Bone	115 (35)	212 (53)	−97	−135 to −60	0.00005	0.0009	2.0	1.0 to 3.0
Glut Max	244 (134)	461 (170)	−217	−354 to −81	0.004	0.009	1.3	0.4 to 2.3

*Note:* Measurement sites: ADL = Midpoint of adductor longus muscle belly, ADL Origin = Adductor longus origin at the pubic bone, Pubic Bone = Pubic bone just adjacent to the symphysis joint, Pubic Symphysis = Anterior part of the symphysis joint, Iliopsoas = Iliopsoas tendon just distally to the inguinal ligament, Glut Max = Midpoint of the gluteus maximus muscle, Elbow = lateral elbow condyle contralateral to the index side.

Abbreviations: Adj. *p*, Benjamini–Hochberg adjusted *p*‐values; CI, confidence interval; Diff, difference; ES, effect size estimated as Cohen's *d*; *n*, number; SD, standard deviation.

**TABLE A4:** Muscle function.

	Symptomatic athletes	Asymptomatic athletes	Symptomatic vs Asymptomatic athletes					
	Mean (SD)	Mean (SD)	Mean diff	95% CI	*p*	Adj. *p*	ES	95% CI
Adduction squeeze test								
Peak torque, Nm/kg	2.4 (0.5)	2.6 (0.5)	−0.2	−0.5 to 0.2	0.27	0.27	0.4	−0.3 to 1.0
RTD_100_, Nm/s/kg	9.9 (6.7)	13.2 (5.3)	−3.3	−7.2 to 0.5	0.09	0.11	0.6	−0.1 to 1.2
RTD_200_, Nm/s/kg	6.3 (3.1)	8.9 (2.9)	−2.6	−4.5 to −0.6	0.01	0.03	0.8	0.2 to 1.5
Abduction press test								
Peak torque, Nm/kg	1.6 (0.3)	1.8 (0.3)	−0.2	−0.3 to 0.005	0.06	0.08	0.6	−0.0 to 1.3
RTD_100_, Nm/s/kg	8.3 (2.5)	10.6 (2.6)	−2.3	−4.0 to −0.7	0.006	0.03	0.9	0.3 to 1.6
RTD_200_, Nm/s/kg	5.6 (1.5)	6.7 (1.6)	−1.1	−2.0 to −0.1	0.04	0.07	0.7	0.0 to 1.3

Abbreviations: Adj. *p*‐value, Benjamini‐Hochberg adjusted *p*‐values; CI, confidence interval; Diff, difference; ES, effect size estimated as Cohen's *d*; Kg, kilogram; Nm, Newton meter; RTD, rate of torque development; s, second; SD, standard deviation.

**TABLE A5:** Between‐group mean differences in pressure pain thresholds on the index side.

Measurement sites	Mean difference	95% confidence interval	Adj. *p*
Adductor Longus Muscle	−110	−176 to −43	0.002
Adductor Longus Origin	−88	−139 to −36	0.001
Iliopsoas	−147	−230 to −64	0.0009
Pubic Bone	−80	−122 to −38	0.0005
Gluteus Maximus	−152	−255 to −49	0.005
Pubic symphysis	−71	−116 to −25	0.003
Elbow	−124	−233 to −15	0.03

*Note:* Measurement sites: ADL = Midpoint of adductor longus muscle belly, ADL Origin = Adductor longus origin at the pubic bone, Pubic Bone = Pubic bone just adjacent to the symphysis joint, Pubic Symphysis = Anterior part of the symphysis joint, Iliopsoas = Iliopsoas tendon just distally to the inguinal ligament, Glut Max = Midpoint of the gluteus maximus muscle, Elbow = lateral elbow condyle contralateral to the index side.

Abbreviation: Adj. *p* = Tukey adjusted *p*‐values.

**TABLE A6:** Within‐group comparisons of Pressure Pain Threshold measurement sites on the index side for the Symptomatic Athletes (*n* = 20).

Comparison: First minus second site	Mean diff.	95% CI	Adj. *p*‐value	Lowest PPT
ADL − ADL Origin	15	−26 to 55	0.92	
ADL − Iliopsoas	−63	−113 to −12	0.007	ADL
ADL − Pubic Bone	20	−25 to 65	0.81	
ADL − Gluteus Maximus	−158	−225 to −92	0.0000001	ADL
ADL − Pubic Symphysis	10	−42 to 61	1.00	
ADL − Elbow	−208	−307 to −108	0.000003	ADL
ADL Origin − Iliopsoas	−77	−132 to −23	0.002	ADL Origin
ADL Origin − Pubic Bone	5	−31 to 42	1.00	
ADL Origin − Gluteus Maximus	−173	−244 to −101	0.0000001	ADL Origin
ADL Origin − Pubic Symphysis	−5	−48 to 38	1.00	
ADL Origin − Elbow	−222	−323 to −122	0.0000007	ADL Origin
Iliopsoas − Pubic Bone	83	21 to 145	0.003	Pubic Bone
Iliopsoas − Gluteus Maximus	−96	−159 to −33	0.0006	Iliopsoas
Iliopsoas − Pubic Symphysis	72	3 to 141	0.04	Pubic Symphysis
Iliopsoas − Elbow	−145	−254 to −36	0.003	Iliopsoas
Pubic Bone − Gluteus Maximus	−178	−260 to −97	0.0000009	Pubic Bone
Pubic Bone − Pubic Symphysis	−10	−44 to 23	0.95	
Pubic Bone − Elbow	−228	−327 to −128	0.0000003	Pubic Bone
Gluteus Maximus − Pubic Symphysis	168	84 to 252	0.000006	Pubic Symphysis
Gluteus Maximus − Elbow	−49	−158 to 59	0.79	
Pubic Symphysis − Elbow	−217	−311 to −124	0.0000003	Pubic Symphysis

*Note:* Measurement sites: ADL = midpoint of adductor longus muscle belly, ADL Origin = adductor longus origin at the pubic bone, Pubic Bone = pubic bone just adjacent to the symphysis joint, Pubic Symphysis = anterior part of the symphysis joint, Iliopsoas = iliopsoas tendon just distally to the inguinal ligament, Glut Max = midpoint of the gluteus maximus muscle, Elbow = lateral elbow condyle contralateral to the index side.

Abbreviations: Adj. *p*‐value, Tukey adjusted *p*‐values; CI, confidence interval; Diff, difference; PPT, pressure pain threshold measured in kilopascals.

**TABLE A7:** Within‐group comparisons of pressure pain threshold measurement sites on the index side for the asymptomatic athletes (*n* = 20).

Comparison: First minus second site	Mean diff.	95% CI	Adj. *p*	Lowest PPT
ADL − ADL Origin	37	−4 to 77	0.10	
ADL − iliopsoas	−100	−150 to −50	0.000006	ADL
ADL − pubic bone	50	5 to 95	0.02	Pubic Bone
ADL − gluteus maximus	−201	−267 to −134	0.0000000004	ADL
ADL − pubic symphysis	49	−3 to 100	0.08	
ADL − elbow	−222	−321 to −122	0.0000006	ADL
ADL Origin − Iliopsoas	−137	−191 to −82	0.00000004	ADL Origin
ADL Origin − Pubic Bone	13	−23 to 50	0.92	
ADL Origin − Gluteus Maximus	−238	−309 to −166	0.00000000003	ADL Origin
ADL Origin − Pubic Symphysis	12	−32 to 55	0.98	
ADL Origin − Elbow	−259	−359 to −158	0.00000002	ADL Origin
Iliopsoas − Pubic Bone	150	88 to 212	0.0000001	Pubic Bone
Iliopsoas − Gluteus Maximus	−101	−164 to −38	0.0003	Iliopsoas
Iliopsoas − Pubic Symphysis	149	79 to 218	0.000001	Pubic Symphysis
Iliopsoas − Elbow	−122	−231 to −12	0.02	Iliopsoas
Pubic Bone − Gluteus Maximus	−251	−332 to −169	0.0000000002	Pubic Bone
Pubic Bone − Pubic Symphysis	−1	−35 to 32	1.0	
Pubic Bone − Elbow	−272	−371 to −172	0.000000005	Pubic Bone
Gluteus Maximus − Pubic Symphysis	249	165 to 333	0.0000000006	Pubic Symphysis
Gluteus Maximus − Elbow	−21	−130 to 87	1.0	
Pubic Symphysis − Elbow	−270	−364 to −177	0.000000001	Pubic Symphysis

*Note:* Measurement sites: ADL = midpoint of adductor longus muscle belly, ADL Origin = adductor longus origin at the pubic bone, Pubic Bone = pubic bone just adjacent to the symphysis joint, Pubic Symphysis = anterior part of the symphysis joint, Iliopsoas = Iliopsoas tendon just distally to the inguinal ligament, Glut Max = midpoint of the gluteus maximus muscle, Elbow = lateral elbow condyle contralateral to the index side.

Abbreviations: Adj. *p*‐value, Tukey adjusted *p*‐values; CI, confidence interval; Diff, difference; PPT, pressure pain threshold measured in kilopascals.
