# Supplementary material for: Pain Sensitivity, Psychological Factors, and Muscle Function in Male Athletes With Long‐Standing Groin Pain and Matched Controls
Source: Scand J Med Sci Sports. 2026 Jul 6;36(7):e70334. doi: 10.1111/sms.70334 (PMC13338664; doi:10.1111/sms.70334)
Supplement: Supplementary file 1 — Table S1: Predetermined order of procedures in the experimental session. [file SMS-36-e70334-s001.docx]

**SUPPLEMENTARY FILE**

Pain sensitivity, psychological factors, and muscle function in male athletes with long-standing adductor-related groin pain and matched controls

Nielsen MF, Ishøi L, Clausen MB, Juhl C, Boudreau S, Graven-Nielsen T, Hölmich P, Thorborg K.

**Content:**

- Predetermined order of procedures in the experimental session
- Description of the clinical examination and classification of groin pain.
- Clinical examination procedure
- Description of radiographic examination
- Muscle function assessment procedure
- Pain Sensitivity
- Pressure pain thresholds
- Description of digital pain drawings using Navigate pain.
- Navigate Pain instruction
- Navigate Pain 2D body charts illustration

**Predetermined order of procedures in the experimental session**

| **Table S1**  **Predetermined order of procedures in the experimental session** | |
| --- | --- |
| **#** | **Procedure / outcome** |
| 1 | Patient demographics assessed with a self-developed baseline questionnaire |
| 2 | Self-reported hip and groin disability assessed with Copenhagen Hip and Groin Outcome Score (HAGOS) |
| 3 | Psychosocial factors assessed with 13 screening questions |
| 4 | Current pain intensity in rest and pain intensity in the preceding week assessed with a numerical pain rating scale (NRPS) |
| 5 | Pain drawings made with Navigate Pain, illustrating usual pain during sport |
| 6 | Pressure Pain Thresholds (PPTs) measured with manual pressure algometry on both the test side (most painful groin) and the contralateral side.  Measurement sites:   1. Midpoint of adductor longus muscle belly – secondary side 2. Adductor longus insertion into the pubic bone – secondary side 3. Iliopsoas tendon just distally to the inguinal ligament. – secondary side 4. The pubic bone just adjacent to the symphysis joint – secondary side 5. Midpoint of adductor longus muscle belly – index side 6. Adductor longus insertion into the pubic bone – index side 7. Iliopsoas tendon just distally to the inguinal ligament. – index side 8. The pubic bone just adjacent to the symphysis joint – index side 9. Anterior part of the symphysis joint 10. The Gluteus maximus muscle – secondary side 11. The Gluteus maximus muscle – index side 12. Lateral elbow epicondyle contralateral to the test side |
| 7 | Pain detection threshold (PDT) and pain tolerance threshold (PTT) measured on the index side at the lower leg using a computer-controlled cuff pressure algometer |
| 8 | Temporal summation of Pain (TSP) measured on the index side at the lower leg using a computer-controlled cuff pressure algometer |
| 9 | Pain detection threshold (PDT) and pain tolerance threshold (PTT) measured on the secondary side at the lower leg using a computer-controlled cuff pressure algometer |
| 10 | Conditioned pain modulation (CPM) measured on the index side at the lower leg using a computer-controlled cuff pressure algometer |
| 11 | Evoked pain intensity (NRPS), bilateral hip adduction peak rate of torque and peak force assessed with the long-lever squeeze test and a handheld dynamometer. |
| 12 | Pain drawings made with Navigate Pain, illustrating evoked pain during the Copenhagen 5 second squeeze test |
| 13 | Evoked pain intensity (NRPS), bilateral hip abduction peak rate of torque and peak force assessed with the long-lever hip abduction press test and a handheld dynamometer. |

**Description of the clinical examination and classification of groin pain.**

Prior to the full clinical examination, patients was screened for potential other severe causes of hip and groin pain. If such causes were identified, the patient was excluded and referred appropriately. All eligible Symptomatic athletes went through the same standardized clinical examination to determine eligibility for participation in experimental testing ^1–3^. The examination of Symptomatic athletes was performed by an orthopaedic surgeon (PH) in the outpatient clinical at Hvidovre hospital.

Based on the clinical examination the subject was categorized with the clinical entities ^2^:

- **Adductor-related groin pain**: Adductor tenderness on palpation AND pain on resisted adduction testing.
- **Iliopsoas-related groin pain**: Iliopsoas tenderness on palpation, above or below the inguinal ligament.
- **Inguinal-related groin pain**: Pain location in the inguinal canal region AND tenderness of the inguinal canal.
- **Pubic-related groin pain**: Local tenderness of the pubic symphysis and the immediately adjacent bone.
- **Hip-related groin pain**: A positive FADIR test AND a positive diagnostic ultrasound guided intra-articular anesthetic hip injection.

The clinical examination was based on the procedure described by Hölmich et al ^1^, which altogether includes palpation of muscle, tendon and bone structures, pain on manual resistance muscle testing, pain on muscle stretching. Additionally, two special orthopedic tests (FABER and FADIR) were performed, and an diagnostic ultrasound guided anaesthetic hip injection was performed if a FADIR test was positive^3^. An intra-articular anesthetic injection has been suggested as a useful examination procedure to distinguish between intra- and extra-articular sources of hip and groin pain^3^. In this study, the intra-articular injection was performed with 8-10 mL 1% lidocaine and did not include corticosteroid. The injection was performed in accordance with the following procedure:

Prior to and 5-15 minutes after administration of the ultrasound guided intra-articular anesthetic hip injection the orthopedic surgeon palpates the distal iliopsoas muscle below the inguinal ligament and medially to the sartorius muscle and performs the FADIR test. During the post-injection assessment, the patient was instructed to indicate the effect of the injection on hip and groin pain using a five-point Likert scale (certain of pain relief; uncertain of pain relief; no change in pain; uncertain of pain worsening; certain of pain worsening) for both iliopsoas palpation and the FADIR test. Inspired by previous studies, a positive response to the injection was defined as “certain of pain relief” whereas we consider “uncertain of pain relief” to “certain of pain worsening” as a negative response ^4^. To blind the patient, only a positive response during the FADIR test was considered a positive intra-articular injection. To limit the risk of false negative findings, a second assessment of iliopsoas palpation and FADIR test was performed if no pain relief during the FADIR test was obtained 5 minutes post-injection. In such symptomatic athletes, patients were instructed to remain in the outpatient clinic for a duration of 15-30 minutes before a second assessment of iliopsoas palpation and FADIR test was performed. Subsequently, the pain response (positive or negative) during only the FADIR test was used by the physiotherapist or orthopedic surgeon to evaluate and classify Symptomatic athletes with hip-joint related groin pain.

**Clinical examination procedure**

1. History taking.
2. Screening tests for ruling out symptoms due to other pathology/problems than hip and groin related
3. Palpation sites for tenderness and known pain:
4. The adductor longus insertion point at the pubic bone
5. The upper insertion of Adductor Magnus
6. The pubic bone just medial and inferior to the pubic tubercle
7. Rectus abdominus insertion/or the Pyramidalis-anterior pubic ligament adductor longus complex (PLAC)
8. Attachment of the Conjoined tendon
9. The superficial inguinal ring
10. Pubic symphysis and adjacent pubic bone
11. The psoas muscle belly superior to inguinal ligament
12. The iliopsoas muscle belly/tendon inferior to inguinal ligament and the insertion of iliopsoas at the lesser trochanter of the femur
13. Muscle resistance and passive range of motion testing for known pain.
14. Adduction of legs against resistance
15. Passive stretching of the adductor muscles
16. Resisted straight and oblique sit-ups
17. Resisted hip flexion in supine
18. The modified Thomas test
19. Special tests:

- Flexion adduction internal rotation (FADIR) test
- Flexion abduction external rotation (FABER) test

1. If FADIR Test is positive 🡪 Diagnostic ultrasound guided intra-articular anesthetic injection

- Palpation of iliopsoas as above (3.8 and 3.9) + FADIR test
- Diagnostic injection
- Walk- around for 10-15 minutes
- Repeated Palpation of iliopsoas as above (3.8 and 3.9) + FADIR test

**Description of radiographic examination**

The radiographic examination consisted of one image with an anterior-posterior pelvic view and one to two images with cross-table lateral view to measure cam and pincer morphology, and to exclude other potential causes of chronic hip and groin pain such as hip dysplasia, stress fracture, osteoarthritis etc. ^5^. This assessment protocol corresponds to the standardized procedure for all patients with chronic hip and/or groin pain seen in the department of orthopedics at Hvidovre Hospital. Findings from the radiological assessment was collected using a standardized case-report form.

The anterior-posterior pelvic view was obtained with the subject standing with equal weight distribution on the legs, hip width distance between the feet and legs internally rotated 15 degrees, so the toes were nearly touching each other. The buttocks of the patient was in contact with the “film”. The pelvis should be centred and positioned symmetrically. The distance from the upper rim of the pubic bone/symphysis joint to the coccyx-sacral joint was between 3-5 cm to obtain near normal inclination of the pelvis. The symphysis joint or the inferior rami of the pubic bone was not covered by the gonad shield.

The cross-table lateral view was obtained with the patient supine and the assessed leg internally rotated 15°. The contralateral hip was flexed and thus lifted out of the x-ray field to avoid interference. The x-ray beam was horizontal and angled 45° from the contralateral side with a distance of 1.2 meters from the transmitter to the film.

Participants were classified with cam (alpha angle >60°^6,7^), pincer (LCEA ≥ 40°^7^ OR LCEA ≥ 35° and a acetabular index angle < 0°^7^), borderline hip dysplasia (LCEA 20-25°) and/or hip dysplasia (a LCEA < 20° or Acetabular index angle > 13°^8^).

From the cross-table lateral view and the anterior-posterior pelvic view the following hip joint-related radiographic findings as described in Lisbon agreement^6,9,10^ were evaluated:

- **Alpha Angle** was defined as the angle between 1) a line from the center of the femoral neck to the center of the femoral head, and 2) a line from the center of the femoral head to the point where the femoral head-neck junction extends beyond the margin of the circle ^7^.
- **Lateral Center Edge Angle** was defined as The angle between 1) a vertical line through the femoral head center and 2) a line between the femoral head center and the lateral sourcil of the acetabulum, as defined by Wiberg.
- **acetabular index angle** was defined as the angle between a horizontal line and a line through the most medial point of the sclerotic zone of the acetabulum and the sourcil ^6^.
- **hip joint space width** was defined as The distance between the femoral head and the lateral sourcil at the acetabulum ^6^.
- **cross-over sign** was defined as the anterior wall contour intersects and becomes lateral to the posterior wall contour. ^6^
- **posterior wall sign** was defined as the posterior acetabular wall projection is medial to the femoral head center projection^6^.
- **Ischial spine sign** was defined as the ischial spine is visible medially to the pelvic rim^6^.

From the anterior-posterior pelvic view, the following pubic-related radiographic findings as described by Serner et al^11^ were evaluated:

- **Bone lucency** “A clear area of decreased attenuation compared to the surrounding bone, which corresponds to an erosion-like configuration and/or cyst.”
- **Erosion-Like Configuration (ELC):** “Irregularities of the cortical bone surface, potentially accompanied by loss of the adjacent trabecular bone.”
- **Superior/Central ELC**: ELC at the superior two thirds of the joint surface
- **Inferior ELC**: ELC at the lower third of the joint surface - “If the entire lower half was considered to have an erosion-like configuration, both of the above were scored as positive"
- **Cysts** “Areas of bone lucency with a sclerotic rim inside the trabecular bone compartment, without accompanying cortical bone surface irregularity.”
- **Proliferation**: “Clear osteophyte outgrowths at the joint margins or within the articular space.”
- **Superior proliferation**: "This can be considered “bone spurs” or classified as “pubic beaking” when bilateral. Well rounded (smooth) bumps at the superior aspect, even if asymmetrical in size, were not considered proliferation. For superior proliferation, the “sharpness” of the superior bone corner angle was used for assistance with angles higher than 90 deg. (obtuse angle) considered “rounded” and scored negative/absent, whereas angles lower than 90 deg. (acute angle) were considered “sharp” and scored as positive/present."
- **Central proliferation**: “Proliferation at the central portion of the articular space.”
- **Inferior proliferation**: "Similar considerations as superior proliferation."
- **Fragmentation** “Clear loose fragment(s) within the symphyseal joint space, or at the inferior medial margin of the pubic bone.”
- **Central fragmentation**: “Clear loose fragment(s) within the symphyseal joint space"
- **Inferior fragmentation**: “Clear loose fragment(s) at the inferior medial margin of the pubic bone.”
- **Sclerosis** “A clear area of increased attenuation of the subchondral bone compared to the surrounding bone, corresponding to an area of increased bone density.”
- **Pubic Symphysis Joint Space Width** “Symphyseal joint space measured in millimeters at the narrowest point of the joint surfaces.”
- **Narrow Joint Space Width**. “if measured to less than 3mm”

**Muscle function assessment procedure**

Muscle function was assessed as maximal isometric and explosive strength during hip adduction squeeze and hip abduction-press tests. Athletes lay supine on an examination bed with legs straight, toes upwards, and hands gripping the bed. In the hip adduction squeeze test, the dynamometer was placed 5 cm proximal to the medial malleoli between the ankles, with the hips abducted ~15°.^12^ In the abduction press test, the dynamometer was fixated 5 cm proximal to the lateral malleoli using a rigid belt, ensuring matched hip abduction angle. For each test, athletes performed one warm-up trial at 50% and one at 100% of perceived max effort, followed by at three valid trials at 100% effort. They were instructed to squeeze or press as fast and hard as possible against the resistance and hold it for 5 seconds.

**Pain Sensitivity**

For assessment of pain sensitivity, athletes lay supine on an examination bed, with support under the knees, and the headrest in a comfortable position.

**Pressure pain thresholds**

For symptomatic athletes, PPTs were assessed on the least painful side before the most painful side. For asymptomatic athletes, the side order was randomized. PPTs were assessed bilaterally at the palpatory middle part of the adductor longus muscle belly (ADDUCTOR LONGUS MUSCLE ), the adductor longus muscle origin at the anterior surface of the superior pubic rami (Adductor longus Origin), the iliopsoas tendon at the point just distally of the inguinal ligament and medial to the rectus femoris and sartorius and lateral to the femoral nerve (Iliopsoas), the pubic bone lateral to the symphysis joint (Pubic Bone), the gluteus maximums at the midpoint between the sacrum and trochanter major (Gluteus Maximus). PPTs were assessed unilaterally at the anterior part of the symphysis joint (Pubic Symphysis) and at the lateral elbow epicondyle (Elbow) contralateral to the most painful side (symptomatic athletes), or second side ( asymptomatic athletes).

**Description of digital pain drawings using Navigate pain.**

The symptomatic athletes reported the quality, intensity, and distribution of usual groin pain during sports activities and evoked groin pain during the hip adduction squeeze test by drawing on high-resolution digital 2D body charts in the web-based application Navigate Pain (Aglance Solutions, Aalborg Denmark). The app was accessed through Google Chrome on an Apple iPad (6. Generation) (Apple Inc., USA) and the drawings were made with an Apple Pencil (Apple Inc., USA). The body charts included front, back and side views, and the app allowed for zooming to improve accuracy. Athletes were instructed to first draw on the front view, and afterwards draw on the other views, using a standardized and scripted instruction. Accordingly, athletes were allowed to draw pain outside of the groin, if they felt it was relevant for their pain experience. Pain was drawn using 11 different quality descriptors (Pain, Dull/Aching, Burning, Throbbing, Stabbing, Tingling, Electric, Numbness, Cold, Itchy, Other), and the 3 intensity levels mild (NRS 0-4), moderate (NRS 5-7) or intense (NRS 8-10). Each descriptor and intensity were drawn with a unique colour and athletes were allowed to create an overlap between descriptors and intensities by drawing with more than one colour on the same area. Pain drawing overlays were used to visualize the pain distribution of all symptomatic athletes in a single figure, with a colour scheme to show the relative frequency of each drawn pixel.

**Navigate Pain instruction**

*Outcome assessor:*

*“We would like to know more about where you experience your pain and how this feels. This will be done using this iPad using this pen to produce two digital pain drawings. Before we get started, I will go through the process for you.”*

*“The two drawings should illustrate your usual groin pain during physical activity or sports and your pain during a test, which we perform later in this session.”*

*“You have to start by drawing on the front of the digital body schema. If it is relevant to you and your pain experience, you should also draw on the right, left or back of the digital body schema. "*

*“Please use the pen to first select pain or a discomfort quality descriptor and then the intensity of that descriptor. Using the pen draw as accurately as possible and to the best of your ability on the digital body schema. When you draw ensure to color in the areas of pain and discomfort completely. Avoid using circle outlines or cross-marks to draw your pain.”*

*“Now, I would like you to draw the first drawing of your current groin pain, here and now, when you are at rest.”*

*Patient:*

*Asks any questions, comments, and makes the first drawing*

*Outcome assessor:*

*“Thank you, now we move on to the next tests of your pain mechanisms before the last drawing which is after a muscle function test.”*

*[Performs other data collection.]*

*Outcome assessor:*

*“Now we are going to test how hard you can squeeze your legs together.”*

*[Test instruction of the Copenhagen 5-Second-Squeeze test]*

*"After the test, I would like you to draw the final pain drawing, which should illustrate the pain experience you had during the test."*

*[Performs the Copenhagen 5-Second-Squeeze test]*

*“Now, I would like you to draw where you experienced groin pain during the squeeze test. Remember to draw as accurately as possible and to the best of your ability on the digital body schema. When you draw ensure to color in the areas of pain and discomfort completely. Avoid using circle outlines or cross-marks to draw your pain.”*

**Navigate Pain 2D body charts illustration**


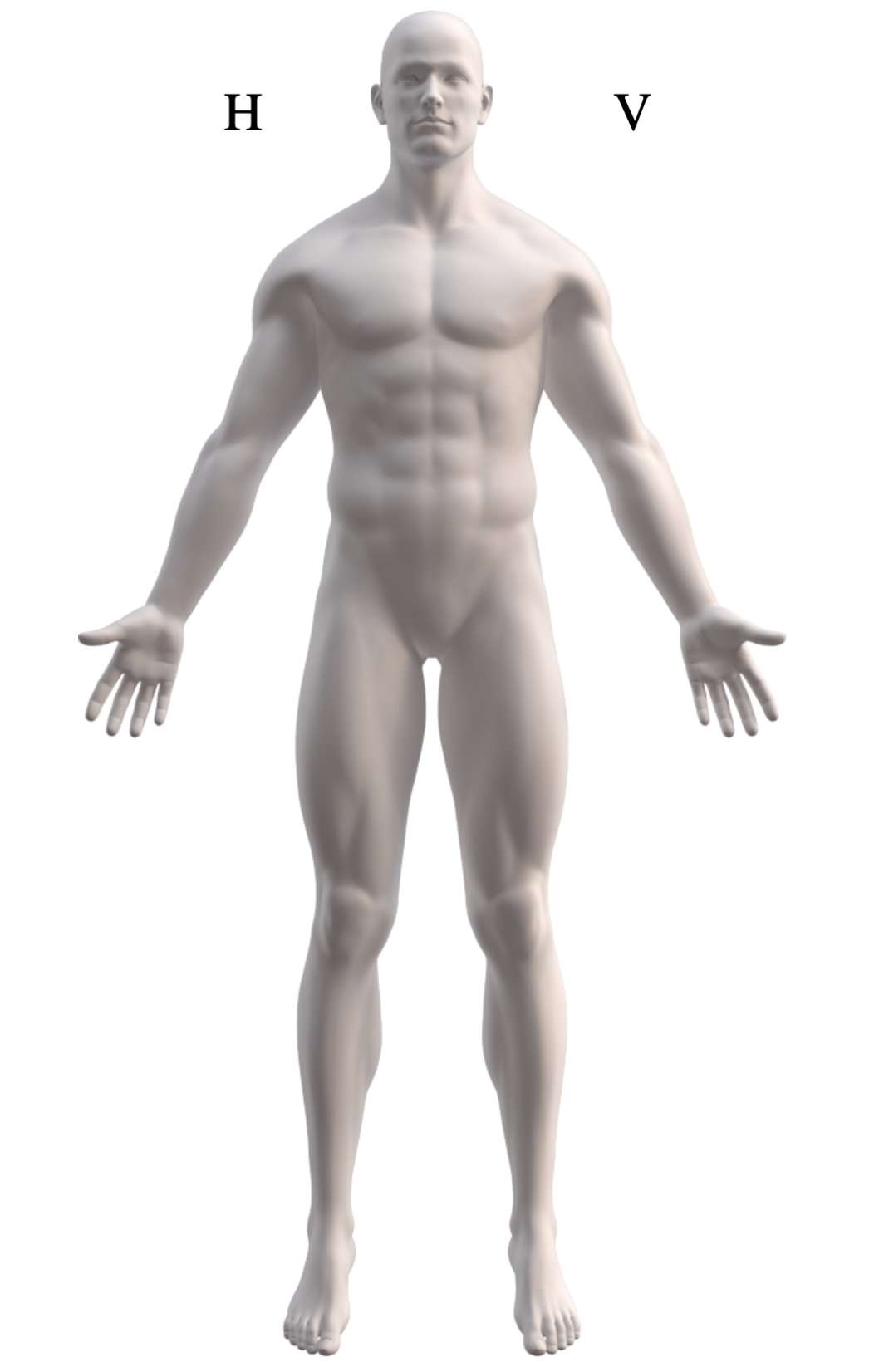

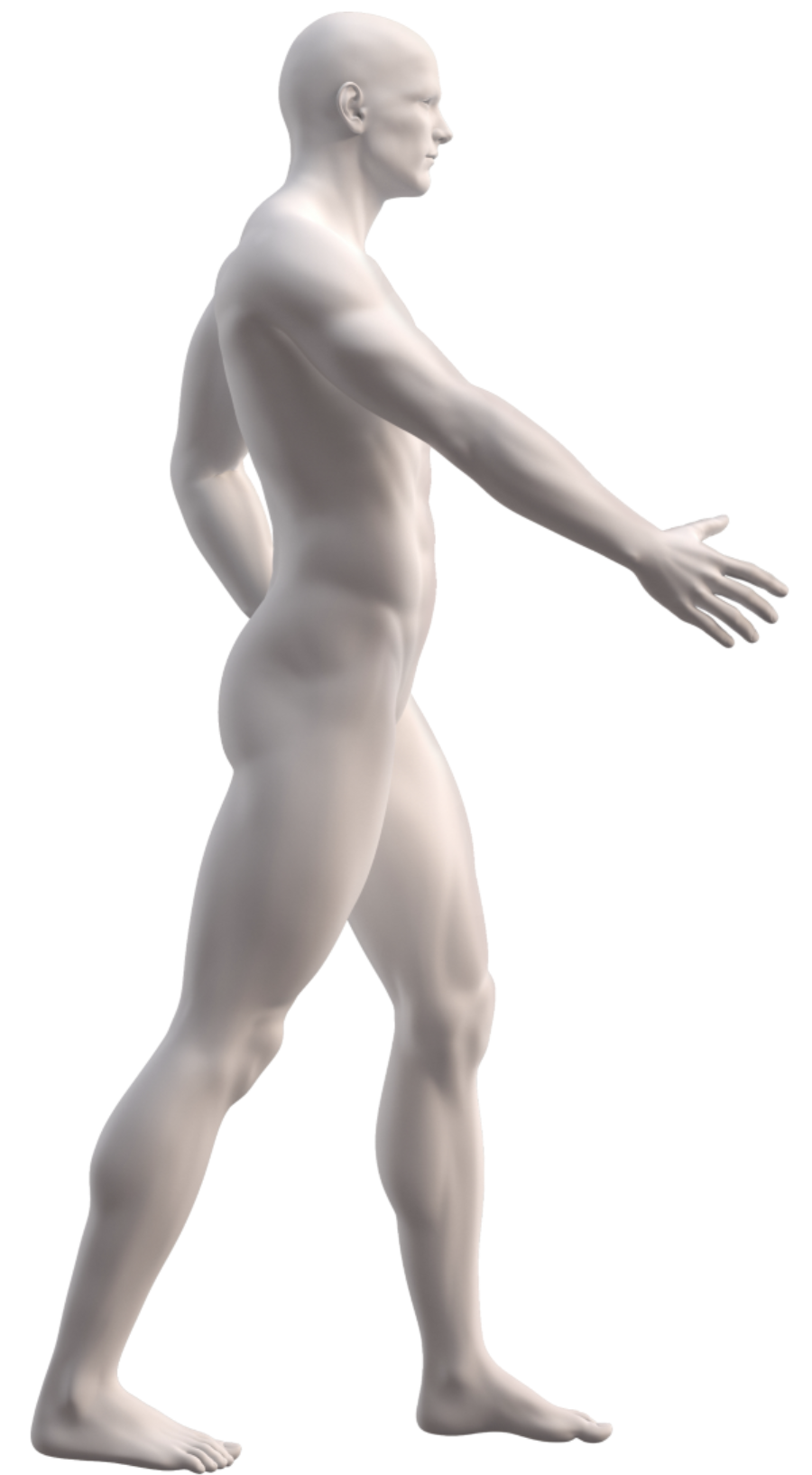

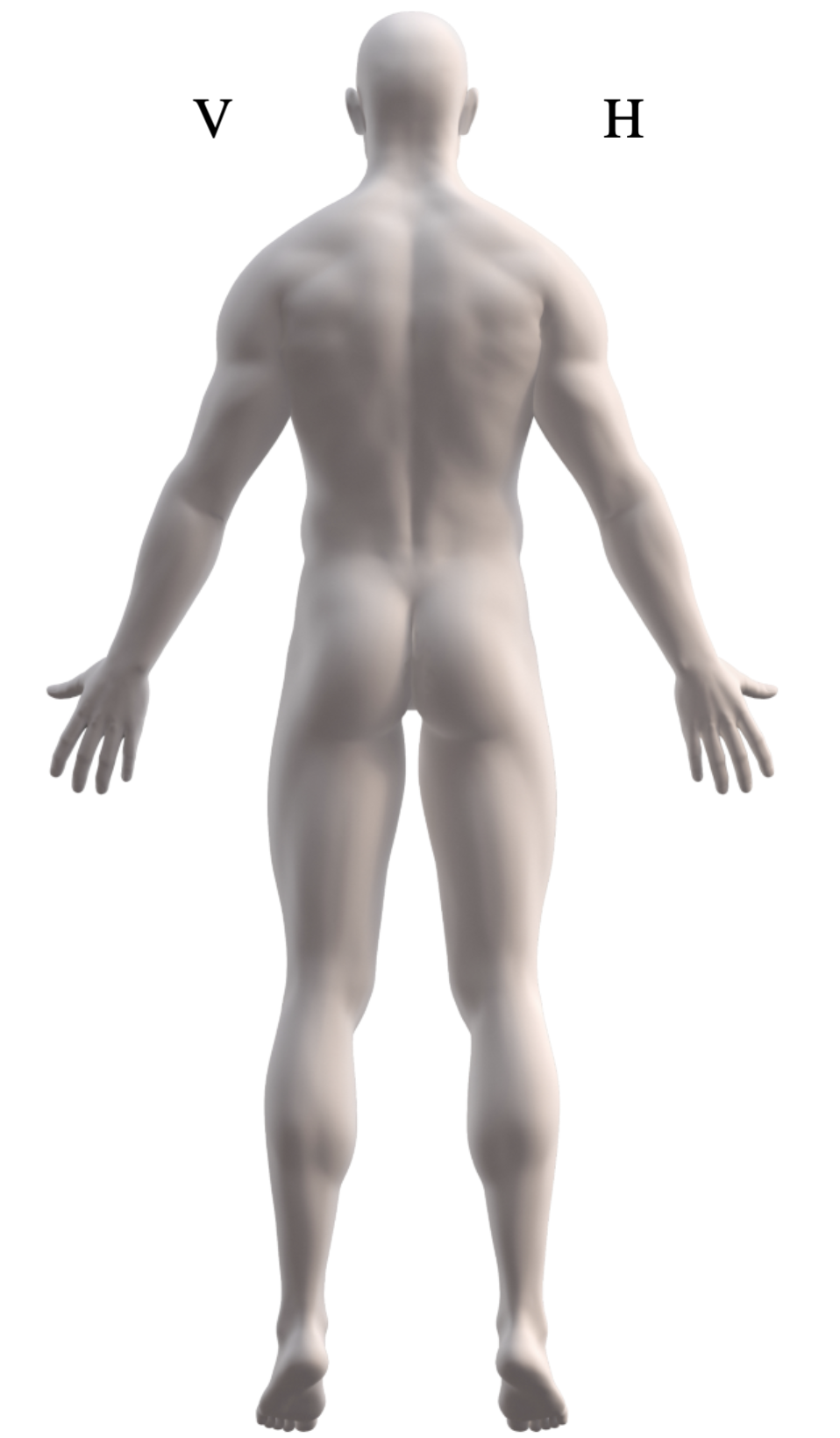


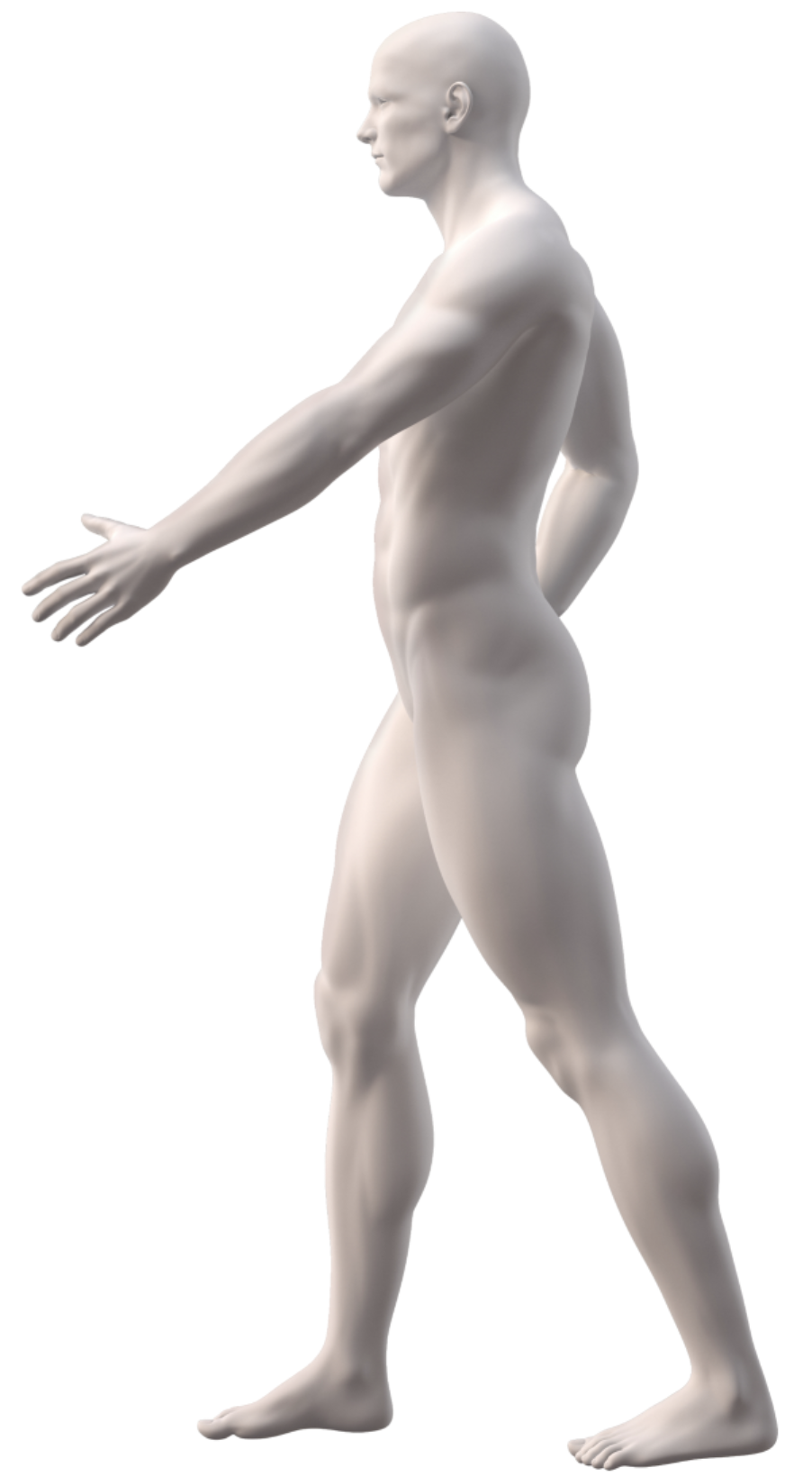


**Description of the Copenhagen Hip And Groin Outcome Score (HAGOS)**

HAGOS is a patient reported outcome measure (PROM) designed to measure hip- and groin-related disability in a young and active populations and recommended for studies on athletes with groin pain^13–15^. HAGOS consists of 37 items on the subscales pain, symptoms, physical function in daily living (ADDUCTOR LONGUS MUSCLE ), function in sport and recreation (Sport), participation in physical activities (PA), and quality of life (QoL). Each item is answered on a five-point Likert scale with a corresponding score of 0-4. Each subscale is summarized in a score ranging from 0 (extreme symptoms) to 100 (no symptoms).^15^. HAGOS has adequate content,^21^ and construct validity,^21^ good test-retest reliability (intraclass correlation coefficients across subscales range from 0.82 to 0.92), is responsive (changes in subscales correlate with Global Perceived Effect),^21^ and can discriminate between players with and without hip and groin pain.^24–26^ HAGOS was recently revised from 37 to 30 items based on evaluation of the structural validity.^27^ We report revised HAGOS scores.
